# Thrombus-specific/responsive biomimetic nanomedicine for spatiotemporal thrombolysis and alleviation of myocardial ischemia/reperfusion injury

**DOI:** 10.1186/s12951-022-01686-1

**Published:** 2022-12-13

**Authors:** Xiaoyu Guo, Ting Hong, Jie Zang, Rongjiao Shao, Xumin Hou, Kai Wang, Weizhuo Liu, Fan Su, Bin He

**Affiliations:** 1 Department of Critical Care Medicine, Shanghai Chest Hospital, Shanghai Jiao Tong University School of Medicine, Shanghai, China; 2 The Institute for Biomedical Engineering & Nano Science, School of Medicine, Tongji University, Shanghai, China; 3 Central Laboratory, Shanghai Chest Hospital, Shanghai Jiao Tong University School of Medicine, Shanghai, China; 4 Centre for Cardiopulmonary Translational Medicine, Shanghai Chest Hospital, Shanghai Jiao Tong University School of Medicine, Shanghai, China

**Keywords:** Myocardial infarction, Ischemia/reperfusion injury, Mitochondrial protection, pH responsive nanomedicine, Platelet biomimetics

## Abstract

Acute myocardial infarction (AMI) is usually caused by coronary thrombosis. However, the short half-life, lack of targetability and inevitable ischemia/reperfusion injury secondary to revascularization, which characterizes tissue plasminogen activator (tPA) limit its thrombolytic efficacy for AMI. To address the targeted and site-specific delivery of tPA, the current study reports the construction of a thrombus-targeting and responsive biomimetic nanoparticle (PTPN) for spatiotemporal treatment of AMI. PTPN was constituted by the thrombus microenvironment- responsive phenylboronic acid (PBA) nanocarrier, antioxidant molecular protocatechualdehyde (PC) and tPA with thrombolytic effect, which were enclosed by the platelet membrane. The thrombus-targeting capability of the platelet membrane enabled the adhesion of PTPN to damaged endothelial cells. The nanoparticle disintegrated under slightly acid condition and re-opened the infarct-related artery during the period of ischemia. Sequentially, ROS induced by blood reperfusion was eliminated by PC released from particle disintegration, and the cardiomyocyte mitochondrial function was protected from reperfusion injury. Therefore, this thrombus-specific/responsive biomimetic nanomedicine provides a spatiotemporal paradigm for AMI treatment with promising clinical translation prospects.

**Supplementary Information:**

The online version contains supplementary material available at 10.1186/s12951-022-01686-1.

## Background

Despite remarkable progress in interventional techniques and treatments, acute myocardial infarction (AMI) which is caused by coronary thrombus occlusion remains a leading cause of mortality worldwide [1]. Rapid revascularization of the infarct-related artery (IRA) is pivotal to improve the clinical outcomes in AMI patients. Thrombolysis remains the first line treatment for AMI treatment, which could be rapidly and easily administered even in undeveloped regions [2]. Tissue plasminogen activator (tPA) is the only FDA-approved thrombolytic medicine for AMI, but it still has many problems [3, 4]. (1) tPA has a short half-life due to its inactivation by plasminogen activator inhibitors (PAI) and clearance by the liver [5]. (2) risk of bleeding and potentially fatal hemorrhage because of repeated administration at short intervals relies on the lack of targets [6, 7]. (3) ischemia/reperfusion injury induced by tPA is associated with adverse cardiovascular events [8].

AMI involves two sequential periods: ischemia and reperfusion, which have different pathological characteristics [9]. The speed of restoration of the blood supply following the onset of AMI is vital to minimize irreversible cardiomyocyte death which is the consequence of severe ischemia and hypoxia [1]. However, the application of tPA for revascularization inevitably triggers secondary ischemia/reperfusion injury due to the production of reactive oxygen species (ROS), which results in cardiomyocytes damage and myocardial fibrosis [8]. At present, there is no delivery strategy to overcome the drawbacks of tPA and tPA-derived problems in AMI treatment at the same time. Therefore, the development of a novel strategy to achieve sequential recovery of blood supply to the heart and scavenging of ROS would bring huge benefits to AMI treatment by protecting cardiomyocyte function from ischemia and reperfusion injury.

**Fig. 1:**
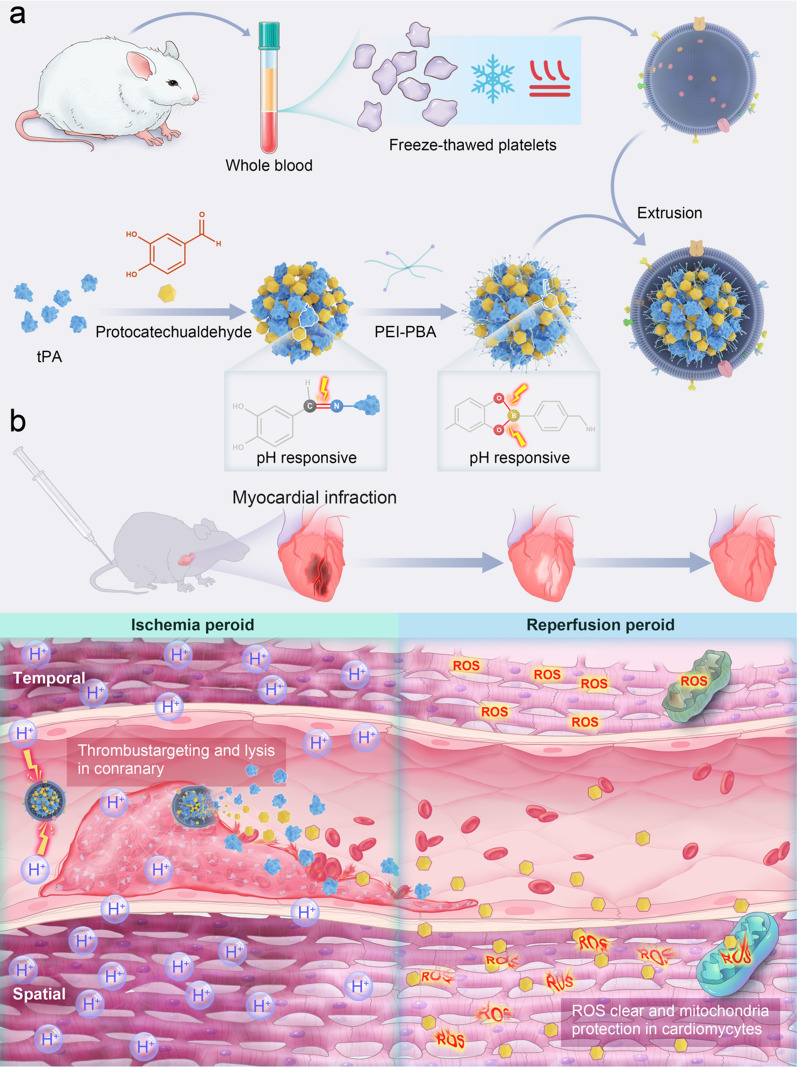
Schematic illustration of PTPN preparation and rescue of ischemia and reperfusion injury. **a** Synthesis method of PTPN. **b** During the ischemia period, PTPN targets the thrombus via platelet membrane and is degraded in response to the acidic microenvironment, thereby releasing tPA to re-open the infarcted artery. During the reperfusion period, antioxidant protocatechualdehyde (PC) is released to clear ROS for cardiomyocyte mitochondria protection

In this work, we constructed a thrombus targeting and responsive nanomedicine via coating the platelet membrane (PTPN) to treat AMI sequentially spatially and temporally. PTPN consists of three components (Fig. 1a): a thrombus-targeting platelet-derived outer membrane and a core containing a pH-responsive phenylboronic acid modified branched polyethyleneimine (PEI–PBA) and protocatechualdehyde (PC) conjugated tPA [10]. Platelet membranes have thrombus-targeting activity due to their content of immunoregulatory proteins and glycoprotein integrins, such as CD42, CD61, and GPIIb/IIIa, which interact with damaged vascular endothelial cells and fibrin [11, 12]. Furthermore, the outer platelet membrane decreases nanoparticle uptake by the mononuclear phagocyte system (MPS) for a prolonged half-life of tPA [13]. The acidic ischemic locale of the AMI microenvironment would precipitate PTPN decomposition to release tPA and re-open the IRA spatially [14]. During myocardial reperfusion, ROS would be scavenged by free PC limiting mitochondrial damage (Fig. 1b). In conclusion, PTPN could be described as ameliorating AMI spatiotemporally by alleviating vessel obstruction and protecting cardiomyocytes mitochondria during both ischemic and reperfusion periods.

## Materials and methods

### Materials and reagents

Materials and reagents were included in Additional file 1.

### Extraction and purification of the PM

Whole blood samples were collected from rats by cardiac puncture with EDTA-treated anticoagulant vacutainer tubes. After that, the whole blood was centrifuged at 200 × *g* for 20 min at 20 ℃. The supernatant fraction was collected and centrifuged at 800 × *g* for 15 min. The white sediments were platelets, which were resuspended with PBS containing EDTA and PGE1 to avoid platelet activation. Then, platelets were lysed by repeated freeze–thaw at − 80 °C and room temperature for 3 times, followed by repeated washes with PBS. Subsequently, platelets were further broken by the ultrasonic cell disruptor (SCIENTZ-950E) for 5 min on ice (100 W, 53KHZ) [11, 15]. Platelet membrane (PM) vesicles were prepared by continuous extrusion through 0.2 and 0.1 μm polycarbonate filters (Avanti® Mini-Extruder set). Finally, total protein of PM was measured by BCA assay.

### Synthesis of PTPN nanoparticles

pH-responsive tPA nanoparticles (TPN) were synthesized as described in our previous work [10]. Briefly, PEI–PBA was achieved by reacting 38.5 mg of PBA, 50 mg of PEI and acid-binding agent trimethylamine in 2 mL of DMSO at 70 °C for 24 h followed by dialysis and lyophilization. PC–tPA was formatted via a Schiff base reaction between the aldehyde group on the PC and the primary amino group on the tPA at 50:1 molar ratio. 29 μL of PC (1 mg/mL) was added into 100 μL of tPA solution (1 mg/mL) at room temperature followed by adjusting pH to 8.0. This reaction was stirred for 1 h and then removed excess PC by ultrafiltration. After that, obtained PC–tPA was stored at 4 ℃ for further use.

Prepared PEI–PBA and PC–tPA at mass ratio of 3:1 were mixed to construct a pH-responsive nanocomplex via boronate ester reaction. Briefly, PC–tPA solution (0.15 mL, 0.12 mg/mL) was added dropwise to PEI–PBA solution (0.15 mL, 0.36 mg/mL) under stirring for 10 min. Meanwhile, mPEG-Dopamine (0.5 mg/mL) was added and continued stirring to obtain the TPN.

Then, TPN and prepared PM were mixed at different protein mass ratios 1:10 for synthetizing PM coated TPN (PTPN) [16, 17]. In brief, TPN (0.5 mL, 0.05 mg/mL) was mixed with PM (0.5 mL, 0.5 mg/mL) and pre-warmed at 37 ℃ followed by continuous extrusion through 0.2 and 0.1 μm polycarbonate filters.

### Characterization of nanoparticles

The particle size and zeta potential of nanoparticles suspended in different pH PBS solutions were determined by dynamic light scattering (DLS) zetasizer (Malvern, Zetasizer 3000). The stability of TPN and PTPN (suspended in PBS or DMEM containing 10% FBS) was evaluated by DLS at 0 h, 12 h, 24 h, 2 d, and 3 d. In addition, transmission electron microscope (TEM) was applied to visualize the morphology of TPN, PM and PTPN.

SDS–PAGE gel with silver staining and Western blot were used to analyze protein profiles of the PM, TPN and PTPN. Briefly, equivalent amounts of protein (4 μg) of PM, TPN and PTPN were separated on a 10% SDS–PAGE gel followed by fixation with ethanol and acetic acid. Subsequently, the gels were incubated with silver staining sensitizer for 2 min and then with silver nitrate for 10 min. Lastly, the gel was incubated with chromogen solution and terminated with stop solution. Similarly, SDS–PAGE gel after electrophoresis was transferred to the 0.22 μm polyvinylidene difluoride membrane (PVDF) and incubated with anti-CD34, anti-CD61, and anti-CD42b overnight. After that, the PVDF membrane was incubated with horseradish peroxidase (HRP)-conjugated secondary antibodies and detected by electro-chemiluminescence (GE, Amersham Imager600).

### Cell model

Primary cardiomyocytes were isolated and cultured according to a previously established method [18]. Briefly, hearts harvested from the neonatal Sprague–Dawley (SD) rats were serially digested with 0.1% trypsin. After that, single-cell suspension was added into cultural dish to remove the fibroblasts by differential attachment for 1 h. The purity of primary cardiomyocytes was confirmed as > 80% by immunofluorescence staining of α-actinin (Additional file 1: Fig. S1) [19]. Human Umbilical Vein Endothelial cells (HUVEC) were purchased from ATCC.

The oxidative stress cell model induced by hypoxia and H_2_O_2_ was used in this work. Cell were cultured in low glucose serum-free DMEM in the hypoxic chamber with 1% O_2_ for 12 h [20]. To mimic the oxidative stress induced by ischemia/reperfusion, cells were treated with 500 μM H_2_O_2_ for 2 h [21]_._

### Animal model

Male ICR mice and SD rats aged 6–8 weeks were purchased from Shanghai JSJ Laboratory Animal Co., Ltd (Shanghai, China). All experimental procedures and animal studies were approved by the Animal Ethics Committee of Shanghai Chest Hospital (Approval number: KS(Y)21,357).

Establishment of the MI rat model: Rats were anesthetized by intraperitoneal (i.p.) injection of 2% paraformaldehyde sodium, followed by endotracheal intubation with 16G cannula (tidal volume = 8 ml/kg, frequency = 80/min) [22]. Then, the chest was opened after blunt dissection of the pectoralis major muscle and pectoralis minor muscle. A 2.5 mm × 2.5 mm filter paper saturated with 15% FeCl_3_ solution was placed on the left anterior descending coronary artery (LAD) for 5 min to induce coronary thrombosis. Finally, the filter paper was removed, and the incision was sutured layer by layer.

Establishment of the femoral artery thrombosis rat model: After anesthesia, the left femoral artery of the rat was exposed and separated from the femoral vein and nerve. A 2.5 mm × 2.5 mm filter paper saturated with 15% FeCl_3_ solution was placed on the femoral artery [23].

Establishment of the carotid artery thrombosis model: ICR mice were used to establish carotid artery thrombosis for live imaging analysis. Briefly, after anesthesia with 0.5% paraformaldehyde sodium and removal of the cervical hair with depilatory cream, the carotid artery was exposed under stereomicroscope (SZX7, Olympus) and covered with a 1 mm × 1 mm filter paper containing 15% FeCl_3_ solution for 5 min [23].

### Safety evaluation

Cell viability was measured by Cell Counting Kit-8 (CCK-8) [24]. In detail, HUVEC were seeded into 96-well plates at a density of 5000 cells per well for 12 h. After that, the medium was replaced with 100 μL of DMEM containing different concentrations (0, 100, 200, 500 and 1000 μg/ml) of tPA, TPN, PM and PTPN for 6, 12, 24 and 48 h. Then, 10 μL of CCK-8 regent was added into each well and incubated for 1 h. Absorbance at 450 nm was measured using the microplate reader (ThermoFihser, VarioscanLUX), and the cell viability was calculated using the following equation:

$${\text{Cell viability}}~(\% )~ = \frac{{{\text{Absorbance}}~({\text{experimental}}~{\text{group}}) - {\text{Absorbance}}~({\text{blank}})}}{{{\text{Absorbance}}({\text{ctrl}}~{\text{group}}) - {\text{Absorbance}}~({\text{blank}})}} \times 100$$

Blood routine examination and clotting function assay were applied to evaluate the biocompatibility of the nanocarrier components. Briefly, 500 μL of normal saline, tPA, PM, TPN and PTPN (20 μg/mL) were injected via the tail vein to the SD rats for acute toxicity test. After 24 h, blood was collected from the retro-orbital venous plexus and placed in sodium citrate anticoagulant tubes. Blood routine examination was performed with the automated blood analyzer (Mindray, BC-2800 Vet) and clotting function was detected with the automatic blood coagulation analyzer (Rayto, RAC-120).

Rats were sacrificed at 4 weeks post intravenous administration. Heart, liver, spleen, lung and kidney were fixed in 4% paraformaldehyde for 24 h, dehydrated, paraffin embedded, and Hematoxylin–Eosin (H&E) stained to observe tissue histomorphometry.

### Binding efficiency of PTPN in vitro

To evaluate the PTPN binding efficiency to HUVEC in vitro [25, 26], HUVEC were seeded into a 24-well confocal plate at a density of 2 × 10^4^ cells per well for 12 h. After that, FITC-labeled TPN, DiI-labeled PM or PTPN extracted with FITC-labeled TPN and DiI-labeled PM (500 ng/mL) was added into the medium. Then, cells were moved to the hypoxic chamber with 1% O_2_ for 1 h. Subsequently, HUVEC were counterstained with Hoechst. Finally, binding efficiency was detected by confocal microscopy (ZEISS, LSM 980 with Airyscan2) and flow cytometry (BD FACSCANTO II).

### Thrombus-targeted ability in vivo

A carotid artery thrombosis model was established to assess the thrombus-targeted ability of PTPN. 100 μL of FITC-labeled PTPN or FITC-labeled TPN (20 μg/ml) was injected via the tail vein of the ICR mice. The distribution of nanoparticles was monitored continuously for 3 h using the In Vivo Imaging System (IVIS; PerkinElmer) [27].

### Thrombolysis ability of PTPN in vitro

Rat whole blood was collected from the retro-orbital venous plexus and placed in 1.5 mL microcentrifuge tubes for coagulation at room temperature [8]. After that, thrombus clots were moved to 24-well plates and incubation with 200 μL of ddH_2_O, tPA (10 μg/mL), PM (10 μg/mL), TPN (10 μg/mL), PTPN (suspended in PBS, pH = 7.4, 10 μg/mL) or PTPN (suspended in PBS, pH = 7.4, 10 μg/mL) respectively. Thrombus clots were extracted and photographed at 1 h or 3 h. After that, solutions containing lysed red blood cells were moved to 96-well plate and absorbance were detected at 538 nm.

### Drug release assay in vitro

Rhodamine labeled tPA was used to synthesize PTPN for release assay. PTPN was dissolved in PBS at pH 7.4 and pH 6.4 respectively, then the release of Rhodamine-tPA at different time points was obtained by ultrafiltration (100 kD, 3000 rpm, 10 min) and detected by fluorescence spectrophotometer.

### Evaluation of tPA activity

As tPA mediates plasminogen conversion to plasmin, the activity evaluation of tPA released from PTPN was measured. PTPN was suspended in PBS at pH 7.4 or pH 6.4 (10 μg/mL), respectively. Subsequently, 2 μL of plasmin substrate-AMC, 20 μL lytic PTPN solution and 48 μL of plasmin assay buffer were added into the plate to detect the plasmin activity. Fluorescent signal was captured every 2 min for 20 min at 37 °C at Ex/Em = 360/450 nm by a fluorometry (ThermoFihser, VarioscanLUX).

### Thrombolysis ability of PTPN in vivo

Nnormal saline, tPA, TPN or PTPN (0.1 mg/kg tPA) was administered via the tail vein in rats after establishment of femoral artery thrombosis. After 6 h, the femoral artery was removed, and H&E staining was performed to detect the recanalization of blood vessels. After FeCl_3_ induced AMI in rats, normal saline, TPN or PTPN (0.1 mg/kg tPA) was administered via the tail vein, and after 6 h the heart was harvested for staining.

### Efficacy of PTPN in AMI treatment

CD31 immunohistochemical (IHC) staining and picrosirius red staining were performed to observe pathological changes of heart. In brief, rats in different groups were sacrificed and perfused with PBS, and then with 4% paraformaldehyde. 5 μm paraffin sections were used for histologic and IHC study. For Picro Sirus Red staining, the sections were immersed into picrosirius red solution for 8 min, dehydrated with anhydrous ethanol twice, and dewaxed twice with dimethylbenzene (5 min each).

For CD31 immunofluorescence, the sections were dewaxed and placed in EDTA buffer (pH = 6.0) to repair antigens subsequently washed with PBS for three times. After that, the sections were immersed in 3% H_2_O_2_ solution and protected from light for 25 min at room temperature. After washing with PBS, the sections were blocked with 5% rabbit serum and incubated with anti-CD31 (1:200) at 4 °C overnight. Then, the sections were incubated with Cy3-conjugated secondary antibodies for 1 h at room temperature.

### Mitochondrial morphology of primary cardiomyocytes

Primary cardiomyocytes were seeded into the 24-well confocal plate at a density of 2 × 10^4^/mL and cultured overnight. The medium was replaced with 500 μL of DMEM containing PBS, tPA, TPN or PTPN (500 ng/mL) for 1 h incubation. H_2_O_2_ was added into the cultural medium at a final concentration of 500 μM for 2 h at 37 °C. The medium was then replaced with DMEM containing MitoTracker™ Red CMXRos and Hoechst 38,450 for 30 min at 37 °C. Finally, primary cardiomyocytes were washed with PBS, and photographed with a confocal microscope.

### Mitochondrial membrane potential assay

Primary cardiomyocytes were cultured and stimulated as described above. Then, the cells were incubated with warm DMEM containing a JC-1 probe for 30 min at 37 °C, washed with warm PBS, re-stained with Hoechst 38,450, and photographed.

### ATP production assay

Primary cardiomyocytes were seeded into an opaque white 96-well plate at a density of 5000 cells per well and cultured overnight. The medium was replaced with 100 μL of DMEM containing PBS or PTPN (500 ng/ml) for 1 h, incubated with H_2_O_2_ (500 nM) for 2 h, and equilibrated for 10 min at room temperature. 100 μL of detection reagent was added into each, shaken well, and incubated at room temperature for signal stabilization [28]. Luminescence was recorded by a plate reader (ThermoFihser, VarioscanLUX) at an integration time of 0.5 s.

### Mitochondrial OxPhos protein expression

Oxidative phosphorylation (OxPhos) supplies most energy for eukaryotic cells, which consists of five protein subunits. Protein expression of OxPhos complexes were detected by Western blot with OxPhos Rodent WB cocktail antibody. Other steps of Western blot were the same as described above.

### RNA sequencing and analysis

Primary cardiomyocytes were collected using a scraper and counted. After washing with PBS, cells were lysed with Trizol (1 mL/10^6^ cells) and stored at − 80 °C. The library preparation and sequencing were conducted by the Haplox (China). Differential expression genes (DEGs) were selected by ∣Fold Change∣ ≤ 1.5 with P value < 0.05. GO function annotations and KEGG pathway database were applied to analyze the potential function and pathway of DEGs. Statistical analysis and graphical plotting were performed using R software.

### Statistical analysis

All numerical data are presented as the means ± SEM. Details and methods are included in the figure legends. Differences between two groups were compared with unpaired Student’s T test, and differences between multiple groups were compared by one-way analysis of variance (ANOVA) followed by the Tukey’s multiple comparisons test. P values are noted in each figure and P value < 0.05 was considered statistically significant.

## Results

### Preparation and characterization of PTPN

**Fig. 2:**
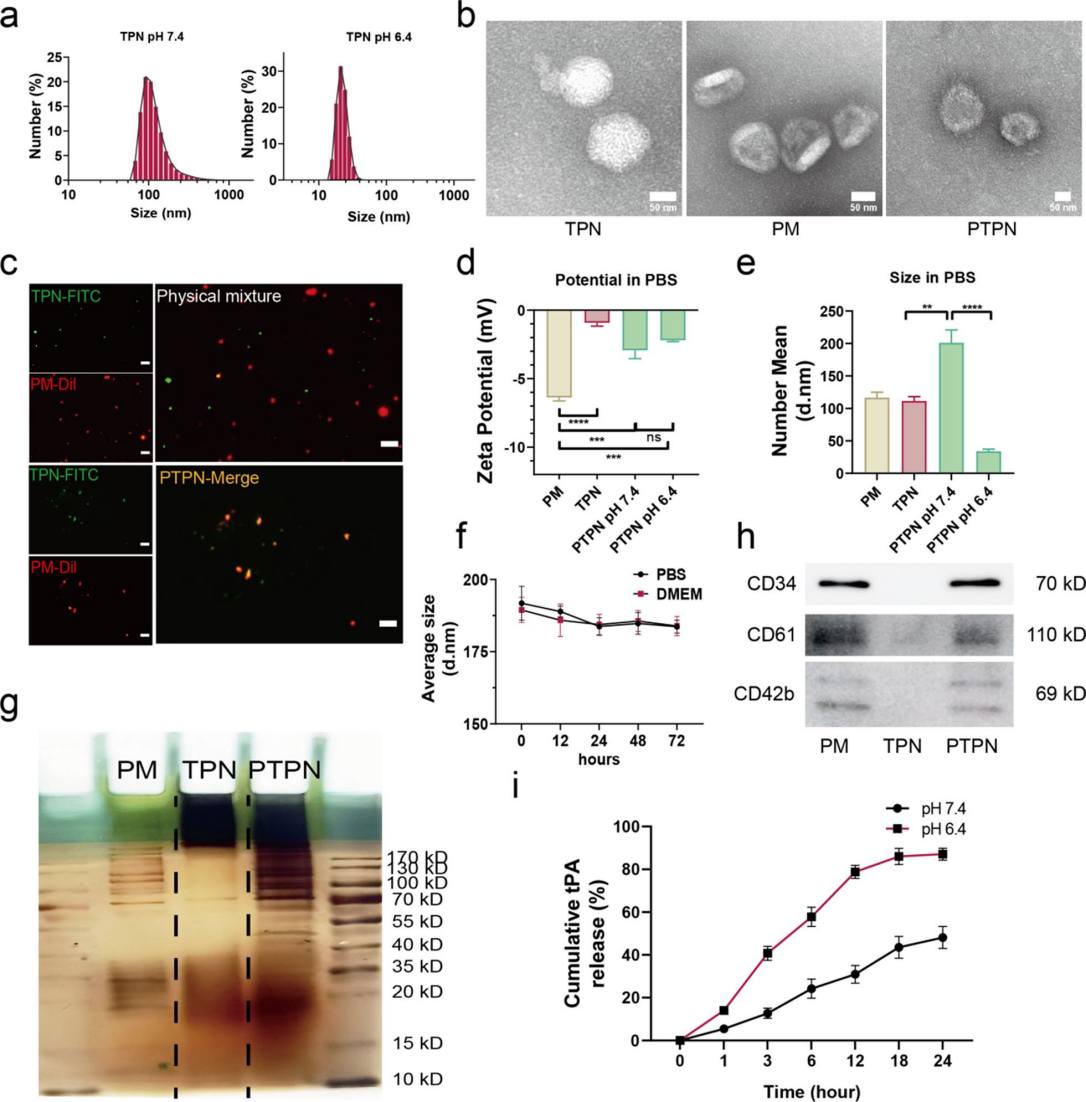
Preparation and characterization of nanoparticles. **a** Hydrodynamic size curves of TPN at pH = 7.4 and pH = 6.4. **b** TEM images of TPN, PM (platelet membrane) and PTPN. Scale bar indicates 50 nm. **c** Confocal fluorescence images of FITC-labeled TPN (green), DiI-labeled PM (red) and membrane-coated PTPN after physical mixture (up) or extrusion (below). Scale bar = 10 μm. Mean zeta-potential (**d**) and hydrodynamic sizes (e) of TPN, PM and PTPN at pH 7.4 and 6.4 (n = 3). **f** Stability of PTPN in PBS and DMEM containing 10% FBS for 72 h (n = 3). **g** Protein profiles of PM, TPN and PTPN in SDS gel with silver staining. **h** Western blot analysis of CD34, CD61 and CD42b in PM, TPN and PTPN, respectively. **i** Release curves of tPA from PTPN in PBS at pH = 6.4 or pH = 7.4 (n = 3). Data are presented as mean ± SEM. Statistical analysis was via one-way ANOVA with GraphPad Prism 8.0; ns: not significant; **p < 0.01; ***p < 0.001 and ****p < 0.0001

The detailed preparation procedure of PTPN was described in the experimental section. In brief, tPA was conjugated to protocatechualdehyde (PC) through schiff bases. Then, pH responsive boronate ester bonds were formed between tPA–PC and PEI–PBA (PBA modified by branched PEI_25k_). As a result, tPA was incorporated into a pH responsive nanocomplex (TPN) to form the core of PTPN. The size of TPN was measured by dynamic light scattering (DLS) and transmission electron microscopy (TEM) in pure water at different pH values. TPN was found to have a diameter of 111.3 ± 11.79 nm with a uniformly dispersed spherical morphology in pure water at pH 7.4 and disintegrated under weakly acidic conditions of pH 6.4 (Fig. 2a and Additional file 1: Fig. S2). Platelet membrane (PM) vesicles of homogeneous hydrodynamic size of 116.5 ± 14.96 nm (Additional file 1: Fig. S3a) and membrane morphology (Fig. 2b) were prepared by differential centrifugation and extrusion according to the method of Zhang et al. [11]. TPN was coated with platelet membrane using a microextruder device (200 nm polycarbonate film) to form PTPN. A TEM image showed the core–shell structure of PTPN (Fig. 2b). TPN was labeled with FITC and PM with DiI to determine the optimal coating ratio for extrusion. FITC–TPN (green) and DiI-PM (red) were dispersed individually under physical mixture, while colocated under extrusion in confocal pictures (Fig. 2c).

Zeta potential values of nanoparticles under different pH conditions were measured by DLS. PM, TPN and PTPN consistently exhibited negative zeta potentials at pH 7.4 and pH 6.4, as a result of negative surface charge of phospholipid bilayer (Fig. 2d). PTPN had a diameter of 201.1 ± 34.38 nm at pH 7.4, decreasing to 34.16 ± 5.206 nm at pH 6.4, indicating substantial acid-mediated disintegration (Additional file 1: Fig. S3b, c, Fig. 2e). PTPN also remained stable in DMEM with 10% fetal bovine serum or in PBS over 72 h (Fig. 2f).

TPN, PM and PTPN were subjected to SDS gel electrophoresis. Silver staining showed a single band for TPN at a molecular mass consistent with that of tPA (60 kD). PM and PTPN showed similar banding patterns, indicating successful coating of PM (Fig. 2g). Specific proteins involved in PM cell adhesion were identified via immunoblotting. PM lysate contained cell adhesive molecules of Integrin beta 3 (CD61), GP-1b (CD42b) and CD34, which were presented in PTPN but not in TPN (Fig. 2h). These results confirmed that PTPN contained both tPA and PM proteins to equip it for its role as a thrombolysis nanoparticle.

Rhodamine labeled tPA was used to synthesize PTPN for release assay. PTPN was dissolved in PBS at pH 7.4 and pH 6.4 respectively, then the release of Rhodamine-tPA at different time points was obtained by ultrafiltration (100 kD, 3000 rpm, 10 min) and detected by fluorescence spectrophotometer. As shown in Fig. 2i, the tPA release profile from PTPN showed 87.08% of the cumulative release rate in acid conditions (pH 6.4) compared with 48.16% in physiological conditions (pH 7.4) at 24 h. Besides, we evaluate the activity of released tPA via a chromogenic substrate assay. Compared with free tPA, the released tPA maintains the activity to convert plasminogen into plasmin (Additional file 1: Fig. S4).

### PTPN safety and cytotoxicity assays

**Fig. 3:**
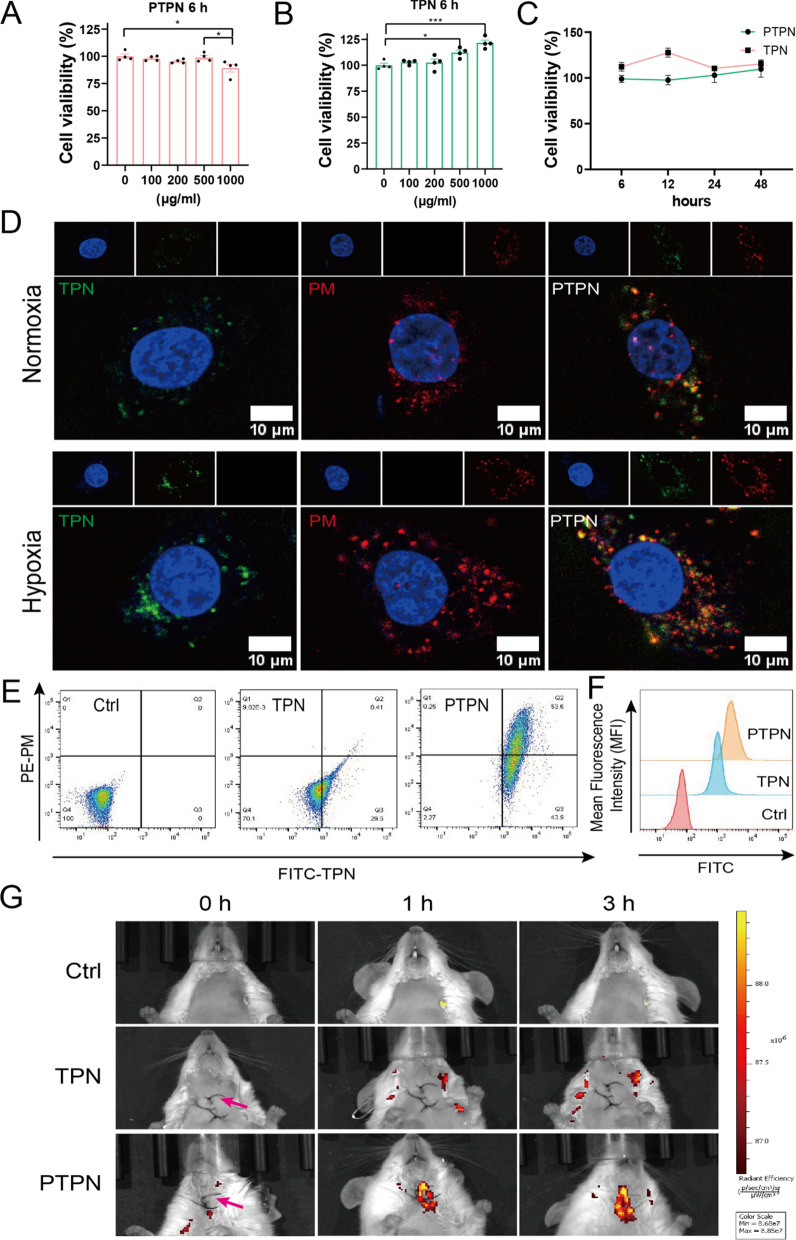
Cell viability and binding capacity of PTPN. **a**–**c** Cell viability of HUVECs treated with PTPN or TPN at different concentrations (**a**, **b**; n = 4; 6 h), or at different incubation time (**c**; 500 ng/mL; n = 3). **d**–**f** Binding capacity of FITC–TPN, DiI-PM and merged-PTPN to HUVECs detected by a confocal microscopy (**d**), and by flow cytometry under normoxia condition (n = 3), displaying representative dot plots (**e**) and histograms of FTIC MFI (**f**). **g** In vivo targeting ability of FITC–TPN and merged-PTPN to carotid artery thrombosis at different time points by detecting FITC signal using IVIS. The red arrow represents the location of the thrombus. Data are presented as mean ± SEM. Statistical analysis was via one-way ANOVA with Bonferroni multiple-comparison correction with GraphPad Prism 8.0; ns: not significant; *p < 0.05; and ***p < 0.001

Safety assessments were conducted both in vivo and in vitro. Endothelial cell injury was an initiating factor in the pathological process of myocardial infarction. HUVECs were incubated in vitro in the presence of various concentrations of tPA, PM, TPN or PTPN. Endothelial viability was not adversely affected by tPA up to 1000 ng/mL or by PM up to 500 ng/mL (Additional file 1: Fig. S5a, b). Similarly, incubation with 500 ng/mL TPN or PTPN for 6 h did not reduce HUVEC viability (Fig. 3a, b). It is noteworthy that both TPN and PTPN (500 ng/mL) had a mildly stimulatory effect on cell proliferation with extended incubation time up to 48 h (Fig. 3c).

Routine blood routine analysis (Additional file 1: Table S1) and coagulation parameters (Additional file 1: Table S2) were used to evaluate the hemocompatibility of the nanoparticles in vivo. No significant alterations were observed at 24 h post intravenous administration of different nanoparticles in rat via the tail vein. Neither the nanoparticles nor their components had any impact on coagulation function, including prothrombin time (PT), activated partial thromboplastin time (APTT), thrombin time (TT) and fibrinogen (FIB). In vivo imaging system (IVIS) was used to track the biodistribution of PTPN. PTPN and TPN showed a tendency to accumulate in liver (Additional file 1: Fig. S6). Heart, liver, spleen, lung and kidney were harvested 4 weeks post-injection of nanoparticles. H&E staining showed no histological abnormalities (Additional file 1: Fig. S7). Thus, PTPN may be considered safe and bio-compatible both in vivo and in vitro.

### Thrombus-targeting ability of PTPN

PM is normally able to target and bind damaged endothelial cells via surface integrin proteins. An in vitro model of damaged endothelial cells resulting from AMI was generated by subjecting HUVECs to hypoxia. PTPN (500 ng/mL, Yellow) showed greater endothelial cell binding capacity than FITC–TPN (Green) under normoxic conditions (Fig. 3d), which was contributed by the adhesion of PE-PM (Red) to HUVECs. Binding of PTPN was accentuated under hypoxic conditions with enhanced localization to HUVECs (Fig. 3d), indicating that hypoxia damaged endothelial cells possessed increased membrane permeability. Flow cytometry analysis revealed that nearly all PTPN and TPN were FITC positive (> 97%; Fig. 3e; Additional file 1: Fig. S8), while the FITC MFI value of PTPN was much higher than that of TPN under normoxic and hypoxic conditions (Fig. 3f, Additional file 1: Fig. S8), suggesting improved cellular internalization in HUVECs. Ferric chloride was used to induce a carotid thrombus in mice and the targeting ability of PTPN was assessed by IVIS. PTPN accumulated at the site of the thrombus to a much greater extent than TPN at 1- and 3-h post-administration (Fig. 3g). These results suggested that PTPN could identify and bind damaged endothelial cells in vitro and accumulated at the site of the target thrombus in vivo.

### PTPN enhanced thrombolytic activity of tPA

**Fig. 4:**
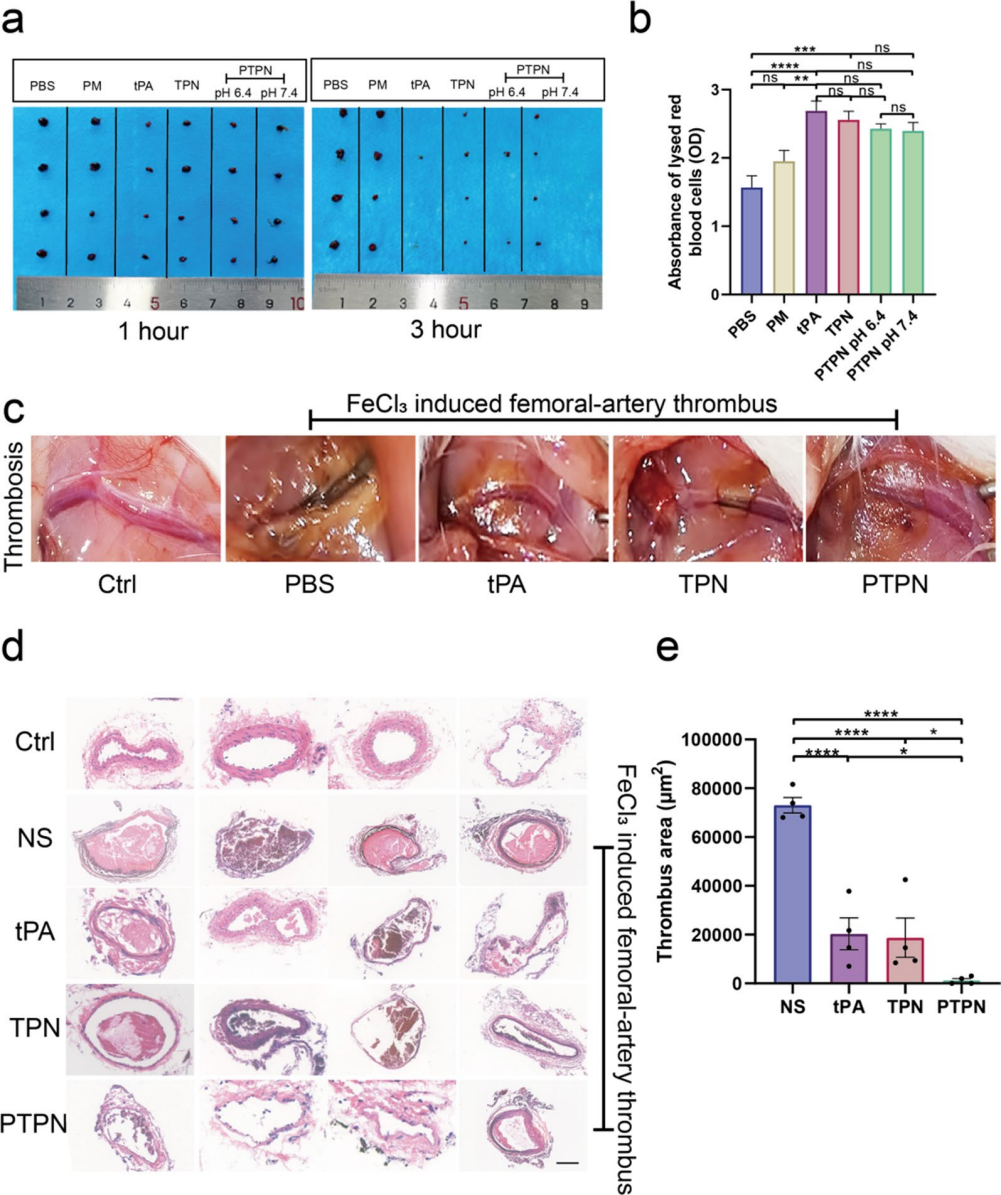
PTPN enhanced thrombolytic activity of tPA. **a** Thrombolytic activity of PBS, tPA, PM, TPN and PTPN (10 μg) at 1 h and 3 h incubation in vitro (n = 4). **b** Quantitative absorbance of lysed red blood cells after 3 h incubation at 538 nm (n = 4). **c** Representative photographs of femoral artery thrombus treated with normal saline, tPA, TPN and PTPN. **d** Images of H&E staining of femoral artery thrombus (n = 4). Scale bar = 100 μm. **e** Quantitative results of thrombus size in femoral artery. NS: normal saline. Data are presented as mean ± SEM. Statistical analysis was via one-way ANOVA with GraphPad Prism 8.0; ns: not significant; *p < 0.05; **p < 0.01; ***p < 0.001and ****p < 0.0001

The thrombolytic ability of PTPN was evaluated both in vivo and in vitro. A thrombus clot was prepared and incubated with PBS, tPA, PM, TPN or PTPN in 24-well plate. As shown in Fig. 4a, thrombus clot was degraded obviously in tPA and PTPN (at pH 6.4) for 1-h incubation. After incubating for 2 more hours, thrombus clot was fully lysed in solution containing tPA component. Degraded solution containing lysed red blood cells was detected via ultraviolet spectrophotometry at 538 nm (Fig. 4b). Femoral artery thrombosis was induced in experimental mice using FeCl_3_ (Fig. 4c) and saline, tPA, TPN or PTPN administered. The infarcted artery was re-opened 3 h after injection with tPA or PTPN to a greater extent than that with TPN. Thus, PTPN had delivered tPA precisely to the thrombus site. H&E staining of the excised tissue showed revascularization after PTPN administration (Fig. 4d, e). In addition, FeCl_3_ was used to induce a coronary thrombosis to mimic AMI. FeCl_3_ administration was seen to produce a large embolus which was successfully thrombolysed by PTPN (Additional file 1: Fig. S9).

### PTPN eliminated ROS and protected mitochondrial morphology

**Fig. 5:**
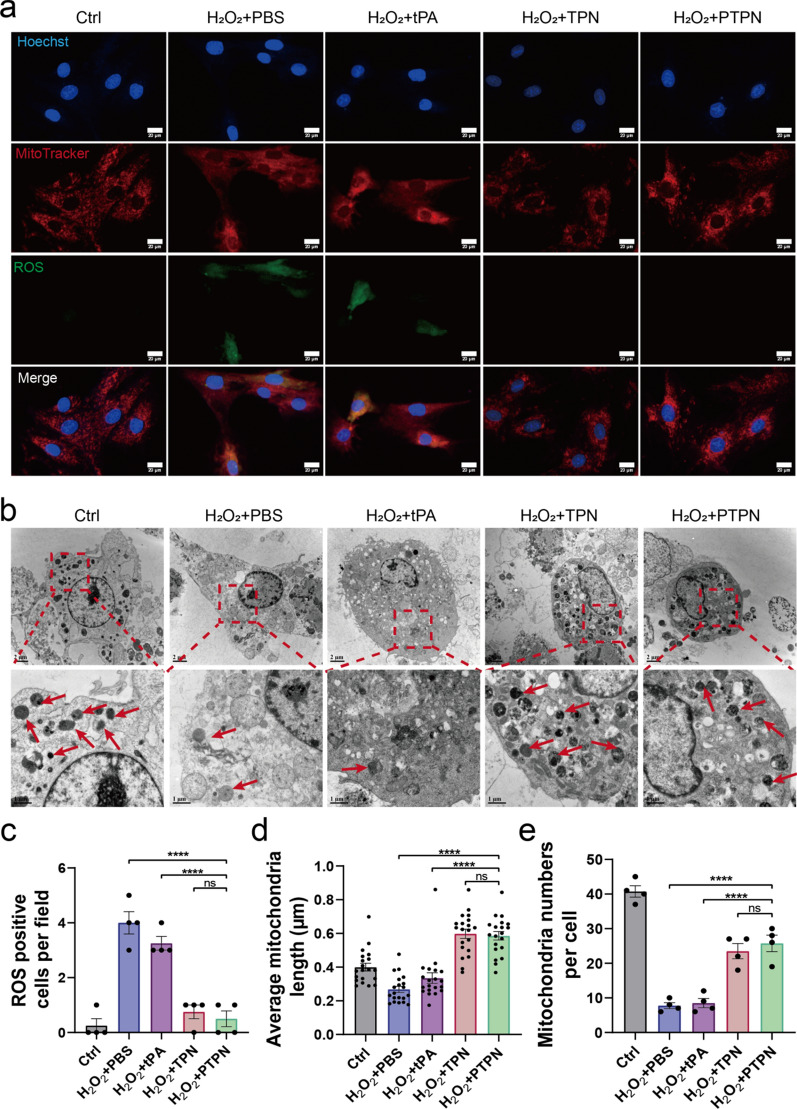
PTPN eliminated ROS and protected mitochondrial morphology. **a** Mitochondrial morphology (MitoTracker, red) and ROS level (DCFH-DA, green) of H_2_O_2_ damaged primary cardiomyocytes treated with PTPN (n = 3; scale bar = 20 μm). **b** TEM images of primary cardiomyocytes; red arrows indicate intact mitochondria. Scale bar = 2 μm in whole field and 1 μm in enlarged scale. **c** Quantitative results of ROS positive primary cardiomyocytes per field in image (**a**) (n = 4). **d** Average mitochondria length of primary cardiomyocytes in TEM (n = 20). **e** Average number of mitochondria of primary cardiomyocytes via TEM (n = 4). Data are presented as mean ± SEM. Statistical analysis was via one-way ANOVA with GraphPad Prism 8.0; ns: not significant; **p < 0.01; ***p < 0.001 and ****p < 0.0001

Ischemia reperfusion injury generates abundant ROS causing severe mitochondrial dysfunction. PTPN contains the antioxidant, PC, for ROS elimination and mitochondrial protection. In the current study, cells were stimulated with H_2_O_2_ to mimic ROS-induced primary cardiomyocyte damage. Then we firstly detected the ROS scavenging ability by PC by flow cytometry (Additional file 1: Fig. S10) and Malondialdehyde (MDA) assay. MitoTracker and DCFH-DA assessment of mitochondrial morphology and ROS level showed disordered mitochondria of abnormal appearance in primary cardiomyocytes (Fig. 5a) and endothelial cells subjected to H_2_O_2_ (Additional file 1: Fig. S11). By contrast, when cardiomyocytes were pretreated with PTPN, normal mitochondrial structure was maintained and ROS levels decreased. As shown in Fig. 5c, DCFH-DA positive cell per field was counted, and PTPN treatment alleviated ROS production compared with free tPA molecules. The number and morphology of mitochondria are the key factors to maintain cardiomyocyte function. TEM images showed that mitochondrial integrity (red arrows) and number decreased following H_2_O_2_ exposure (Fig. 5b), while pretreatment with PTPN protected cardiomyocyte mitochondria. Average length (Fig. 5d) and number (Fig. 5e) of mitochondria was calculated according to TEM images, which proved the mitochondria protective function of PTPN pretreatment.

### PTPN maintained mitochondrial respiratory chain function

**Fig. 6:**
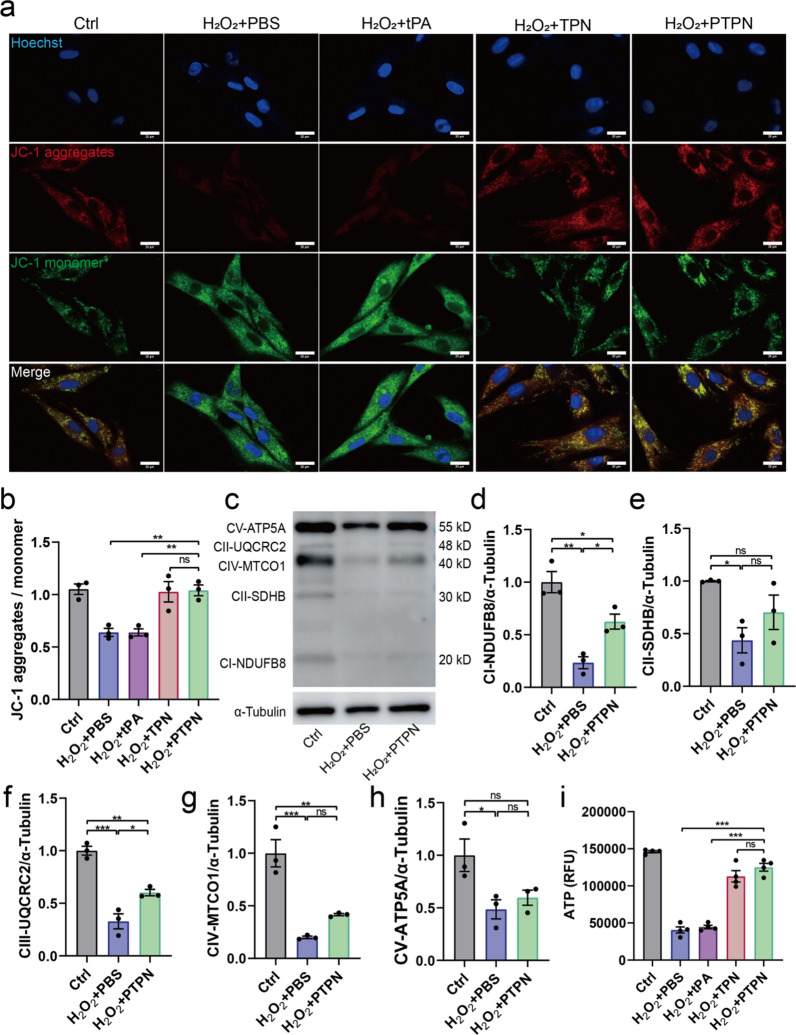
PTPN maintained mitochondrial respiratory chain function. **a** Mitochondrial membrane potential (JC-1) of primary cardiomyocytes treated with PTPN. Red indicated JC-1 aggregates and green indicated JC-1 monomer. (n = 3, scale bar = 20 μm). **b** Quantitative results of mitochondrial membrane potential. **c** Western blot analysis of OxPhos complexes in primary cardiomyocytes treated with PTPN. Relative protein expression of OxPhos subunits: NDUFB8 (**d**), SDHB (**e**), UQCRC2 (**f**), MTCO1 (**g**) and ATP5A (**h**) (n = 3). **i** ATP level of primary cardiomyocytes treated with PTPN (n = 4). Data are presented as mean ± SEM. Statistical analysis was via one-way ANOVA with GraphPad Prism 8.0; ns: not significant; *p < 0.05; **p < 0.01; and ***p < 0.001

H_2_O_2_ treatment obviously decreased the mitochondrial membrane potential (decreased JC-1 monomer, green) in both primary cardiomyocytes (Fig. 6a, b) and endothelial cells (Additional file 1: Fig. S12), suggesting mitochondrial function damage. In contrast, the treatment of TPN or PTPN (but not tPA) significantly increased the mitochondrial membrane potential, restoring the function of mitochondria. Five electron transport chain (ETC) proteins that are responsible for mitochondrial ATP-synthesis, were measured by immunoblots. Exposure to H_2_O_2_ reduced the expression levels of these proteins, which was ameliorated by PTPN (Fig. 6d–h). ATP generated by mitochondria was the major cellular energy source. The result of ATP production (Fig. 6i) demonstrated that PTPN could retain the mitochondria function of cardiomyocytes stimulated by H_2_O_2_.

### Therapeutic mechanism of PTPN in mitochondria protection

**Fig. 7:**
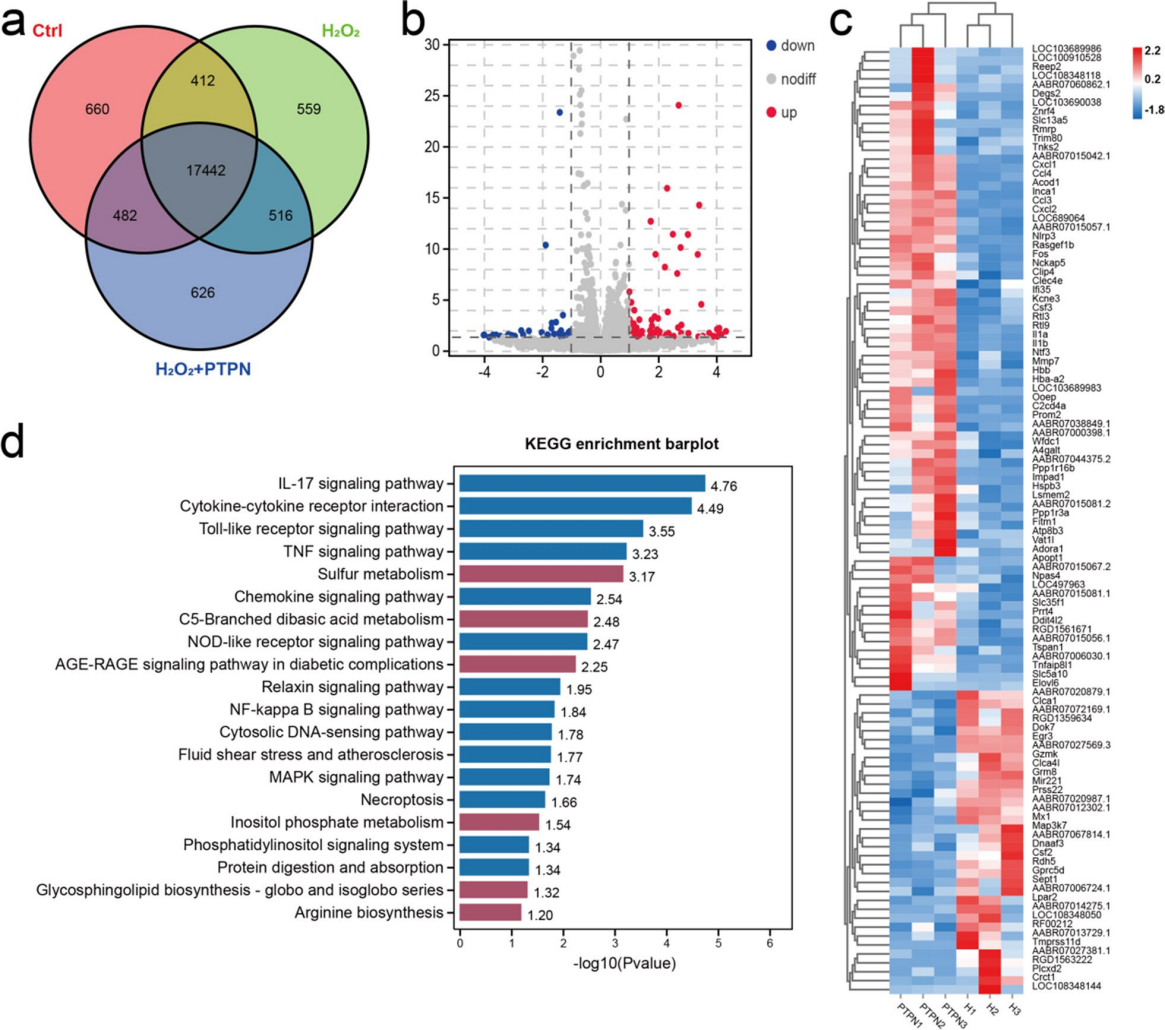
Mechanistic study of PTPN in myocardial infarction treatment by next generation sequencing. **a** Venn diagram of all genes detected in Ctrl, H_2_O_2_ + PBS and H_2_O_2_ + PTPN conditions. (n = 3 biologically independent samples). **b** Volcano map of differentially expressed genes (DEG; adjusted p < 0.05; fold change ≥ 2) between H_2_O_2_ + PBS and H_2_O_2_ + PTPN groups, red represented up-regulated genes and blue represented down-regulated genes. **c** Heatmap of DEGs from H_2_O_2_ + PBS and H_2_O_2_ + PTPN groups. **d** KEGG enrichment barplot of DEGs between H_2_O_2_ + PBS and H_2_O_2_ + PTPN groups

Primary cardiomyocytes pre-treated with PTPN or PBS were exposed to H_2_O_2_ followed by RNA-sequencing. Similar levels of homogeneity were found for controls, PBS + H_2_O_2_ and PTPN + H_2_O_2_ (n = 3), ensuring the reliability of further analysis (Additional file 1: Fig. S13a). A Venn diagram showed the overlapping gene expression detected among the three groups (Fig. 7a). H_2_O_2_ challenge led to significant changes in RNA profile of cardiomyocytes in vitro which were further altered by PTPN treatment. Differentially expressed genes (DEGs) were illustrated in the Volcano diagram (Fig. 7b) and heatmap (Fig. 7c) for the comparison of cardiomyocytes stimulated by H_2_O_2_ to those pretreated with PTPN (DEGs; adjusted p < 0.05; fold change ≥ 2). In total, there were 120 DEGs including 85 up-regulated and 35 down-regulated. Based on these DEGs, gene ontology (GO; Additional file 1: Fig. S13b) and Kyoto Encyclopedia of Genes and Genomes (KEGG) functional enrichment analysis (Fig. 7d) were performed. The pathways enriched included sulfur metabolism, C5-branched dibasic acid metabolism, ARE-RAGE signaling pathway (diabetic complications), inositol phosphate metabolism, glycosphingolipid biosynthesis (globo and isoglobo series) and arginine biosynthesis (Fig. 7d).

### PTPN alleviated myocardial infarction and promoted angiogenesis

**Fig. 8:**
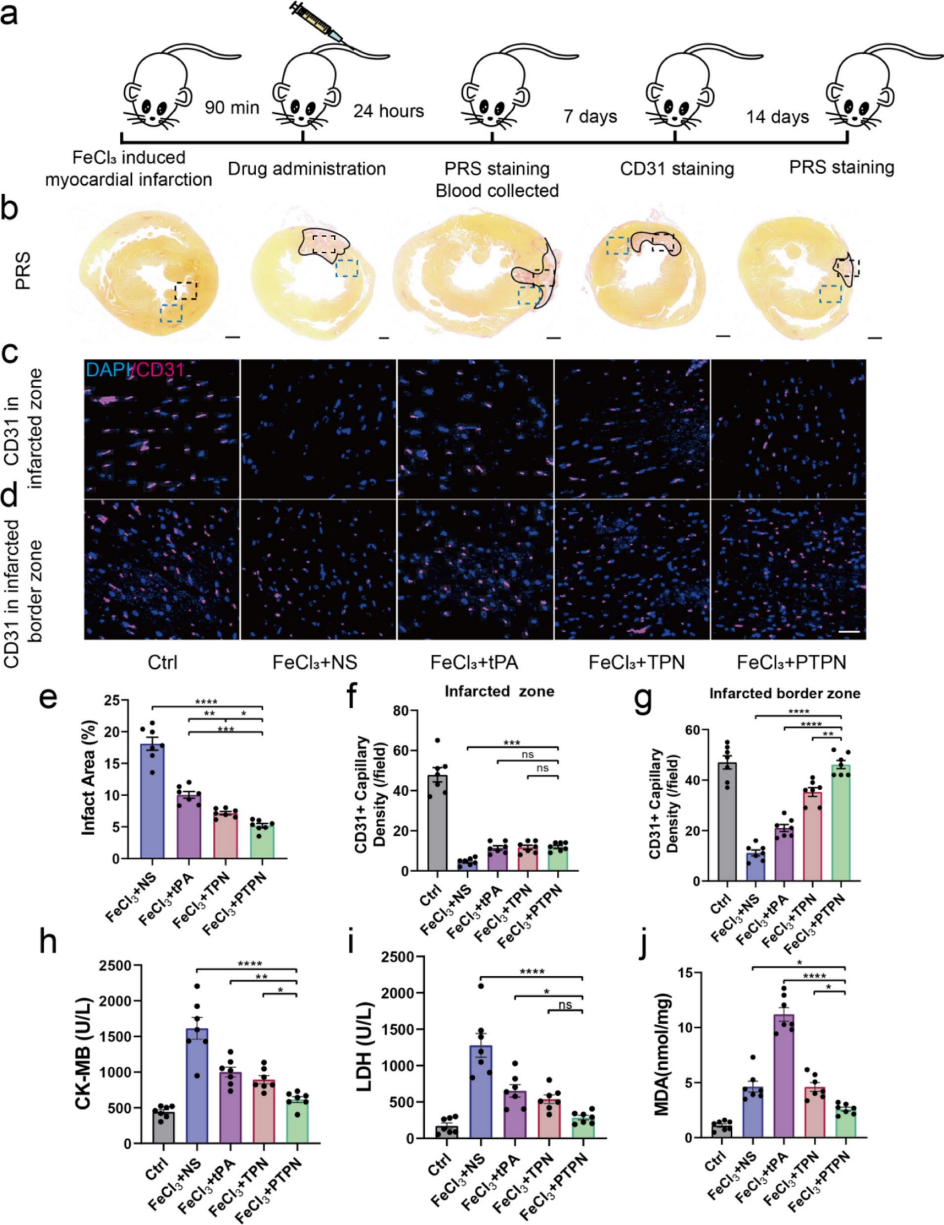
PTPN alleviated myocardial infarction and promoted angiogenesis. **a** Animal experiment timeline. **b** Representative photographs of infarcted area with PRS staining (n = 7). Black lines indicated infarct zone, black frame indicated infarcted zone and blue frame indicated infarcted border zone. Scale bar = 1 mm. **c**, **d** CD31 expression in infarcted zone (**c**) and infarcted border zone (**d**) by immunohistochemical staining with different treatments (n = 7). Scale bar = 20 μm. Quantitative analysis of infarct size (**e**); CD31 + capillary density per field in infarcted zone (**f**) and infarcted border zone (**g**). Serum CK-MB (**h**), LDH (**i**) and MDA (**j**) levels (n = 7). Data are presented as mean ± SEM. Statistical analysis was via one-way ANOVA with GraphPad Prism 8.0; ns: not significant; *p < 0.05; **p < 0.01 and ***p < 0.001

Damage due to both ischemia and reperfusion contributes to poor prognoses in AMI. The use of tPA to remove clots and relieve coronary thrombosis does not, in itself, produce an alleviating effect on ROS-production due to reperfusion. A mouse model of coronary thrombus was induced by FeCl_3_ and subjected to different treatments after 90 min as illustrated in Fig. 8a. Blood was collected after 24 h for the analysis of myocardial enzymes and inflammatory factors. Hearts were harvested after 7 and 14 days to assess degrees of angiogenesis and myocardial fibrosis. Pirus red staining showed a substantial reduction in the infarcted area after PTPN treatment with significantly improved thrombolytic activity compared with other conditions (Fig. 8b, e). Immunofluorescence analysis of CD31 expression showed that PTPN promoted capillary angiogenesis in the infarct border zone (Fig. 8d, g), although not in the infarct zone (Figs. 8c, f). The stimulation of angiogenesis is likely to be the result of ROS elimination by the PC component. Myocardial damage was assessed by release of the blood markers, creatine kinase (CK-MB) and lactate dehydrogenase (LDH). Levels of these markers were increased significantly after MI and alleviated by re-opening of vessels in the current model (Fig. 8h, i). We detected the ROS production after PTPN treatment compared with the platelet membrane coated tPA nanoparticle (PTN@M) without antioxidant capacity via DHE probe. As shown in Additional file 1: Fig. S14, myocardial infarction without reperfusion produced less ROS than free tPA treatment or PTN@M. Once reperfusion occurred, PTPN could significantly decrease the ROS level compared with free tPA, PTN@M, or TPN for ROS scavenger PC. Lipid peroxidation Malondialdehyde (MDA) was determined by treatment of PTPN, which assay is an indicator of ROS induced by ischemia/reperfusion injury (Fig. 8j). We detected the Picro sirus red staining after 14 days (Additional file 1: Fig. S15) which demonstrated the alleviation of myocardial fibrosis by PTPN treatment. In addition, levels of pro-inflammatory factors, including TNF-α and IL-1β, reflected the presence of infarction associated-aseptic inflammation. Levels of TNF-α and IL-1β were reduced by treatment with PTPN (Additional file 1: Fig. S16a, b). In summary, treatment with PTPN promoted neovascularization of the infarct border zone, stimulated angiogenesis in the recovering tissue and reduced fibrosis. These effects are expected to contribute to the recovery of the myocardium from ischemic damage and reduce adverse remodeling of affected tissue.

## Discussion

Formation of a coronary thrombus is the initiatory factor for AMI and timely revascularization of infarct-related arteries is crucial to treatment [18]. Although tPA is recognized as an FDA-approved thrombolytic medicine, its short half‐life in circulation and the potential risk of major bleeding have limited its utility in AMI. Moreover, tPA does not prevent damage caused by ROS that occurs after reperfusion and is associated with poor prognosis [29]. Many small molecules with antioxidant activity have been tested to ameliorate ischemia/reperfusion injury. Indeed, ischemia and reperfusion associated injury has usually been treated separately, whereas the simultaneous administration of antioxidants and restoration of blood supply would be more beneficial. Biomimetic nanotechnology has provided new insights for designing novel thrombolytic drugs by encapsulating compatible multicomponent drugs. Some studies have designed pH-sensitive nanocarriers that deliver thrombolytic drugs or brain-protective drugs with antioxidant small molecules at the same time, and have shown excellent synergistic effects in stroke treatment [8, 30]. tPA and nitroxide antioxidant 4-amino-2,2,6,6-tetramethylpiperidine-1-oxyl was self-assembled into pH-sensitive antioxidant nanoparticles (t-PA@iRNP), which has synergistic effect of thrombolysis and antioxidant for stroke [8]. However, our work differs from previous studies in two main aspects. On the one hand, the co-delivery of thrombolytic drugs and antioxidants has rarely been reported in the treatment of myocardial infarction. On the other hand, we designed a pH-responsive nanocarrier based on catechol-boronate chemistry to deliver thrombolytic tPA. In this work, antioxidant PC was not only used to form an acid-sensitive chemical bond for site specifically release thrombolytic tPA, but also played a sequential antioxidant role in the reperfusion stage of myocardial infarction.

Precise delivery to the specific site of the thrombus is crucial to improve tPA treatment efficiency and reduce side effects. Zhang et al. have produced a systematic description of functional components of platelet membrane vesicles required for cardiovascular therapeutic purposes [11]. In our work, we developed an AMI environment responsive PBA nanocarrier, adjusted from our previous work, designed to disintegrate under slightly acidic conditions and release tPA for thrombolysis [10]. PBA nanoparticles were coated with platelet membrane by extrusion in order to confer thrombus-targeting properties. The resulting PTPN bound and targeted damaged endothelial cells and thrombus both in vitro and in vivo. Complete release of tPA from PTPN was achieved at pH 6.4 in vitro and blocked arteries were re-opened in vivo in multiple animal thrombus models.

Ischemia/reperfusion injury is characterized by ROS overload which causes cardiomyocyte dysfunction and morphological changes, including decreased mitochondrial numbers and membrane potential. There has been a considerable body of research focused on developing anti-oxidative nanoparticles to clear ROS and alleviate ischemia/reperfusion injury [31, 32]. In a previous study, we synthesized polydopamine nanoparticles to scavenge ROS and prevent cell pyroptosis in the treatment of ischemia/reperfusion injury [17]. However, this approach lacked the capacity for restoration of blood supply which would be necessary for treatment of AMI. In addition, previous approaches which have included the systemic administration of anti-oxidative molecules, such as vitamin C, have observed low concentrations of antioxidants in the myocardial locality during clinical trials [33–35]. By contrast, the antioxidant, PC, included in PTPN was delivered specifically and promptly to cardiomyocytes during the reperfusion phase due to the targeting ability of the nanoparticles. Next generation sequencing used during the current study confirmed that mitochondrial metabolic functions of cardiomyocytes was protected by PTPN treatment.

We acknowledge some limitations to the current work. Further experiments should be conducted in a large animal model, such as the pig, in which coronary recanalization may be monitored through the use of angiography. In addition, the mode and speed of PTPN clearance in vivo requires further evaluation.

## Conclusion

In summary, a biomimetic nanoparticle (PTPN) with spatiotemporal activity was rationally designed and engineered for the effective treatment of AMI to increase the efficiency of tPA delivery to the site of infarct. By exploiting different pathological microenvironment characteristics of ischemia and reperfusion, PTPN (i) targeted the thrombus site and featured a prolonged circulating half-life due to its platelet membrane coating; (ii) responded to the acidic microenvironment of AMI and restored the blood supply; (iii) protected mitochondria and cleared ROS to prevent reperfusion injury. Systematic in vitro and in vivo evaluations solidly demonstrated the high efficacy of the engineered PTPN nanomedicine for the spatiotemporal thrombolysis and alleviation of myocardial ischemia/reperfusion injury, in accompany with the detailed mechanism investigation by RNA-sequencing. This spatiotemporally biomimetic nanoparticle offers a systematic and sequential scheme to improve the current treatments for acute myocardial infarction.

## Supplementary Information

**Additional file 1: Figure S1.** The purity of primary cardiomyocytes. **Figure S2.** TEM images of TPN in different pH conditions. **Figure S3.** Hydrodynamic size curves of PM (a), and PTPN in pH 7.4 (b) and pH 6.4 pure water solution (c). **Figure S4:** Activity of free tPA and PTPN to convert plasminogen to plasmin after 24 hour incubation. **Figure S5.** Cell viability of HUVECs treated with tPA (a) and PM (b) at different concentrations. **Table S1.** Routine blood routine analysis. **Table S2.** Coagulation function. **Figure S6.** The distribution of TPN and PTPN in different organs detected by IVIS. **Figure S7.** H&E staining images of organs harvested at 4 weeks post-injection of nanoparticles. **Figure S8.** Binding capacity of TPN, PM and PTPN to HUVECs detected by flow cytometry under hypoxia condition. **Figure S9.** H&E staining of cardiac thrombus with different treatments. **Figure S10.** ROS level of H9C2 under different treatments were detected by flow cytometry and MDA assay. **Figure S11.** Mitochondrial morphology and ROS level of H_2_O_2_ damaged HUVECs treated with PTPN. **Figure S12.** Mitochondrial membrane potential (JC-1) of HUVECs treated with PTPN. **Figure S13.** (a)TPM distribution of primary cardiomyocytes without treatment (Ctrl) or treated with H_2_O_2_ + PBS and H_2_O_2_ + PTPN. **Figure S14.** ROS level after different treatments with DHE staining. **Figure S15.** Fibrosis level after different treatments with PRS staining. **Figure S16.** Serum IL-1β (a) and TNF-α (b) levels in AMI rats with different treatments.

## Data Availability

The data are all available upon request.

## Abbreviations

ANOVA: One-way analysis of variance
APTT: Activated partial thromboplastin time
CCK-8: Cell Counting Kit-8
CK-MB: Creatine kinase
DEGs: Differential expression genes
DLS: Dynamic light scattering
DMEM: Dulbecco’s Modified Eagle Medium
ETC: Electron transport chain
FBS: Fetal bovine serum
FIB: Fibrinogen
FITC: Fluorescein isothiocyanate
GO: Gene ontology
H&E: Hematoxylin–Eosin
HRP: Horseradish peroxidase
HUVEC: Human Umbilical Vein Endothelial cells
IHC: Immunohistochemical
IRA: Infarct-related artery
IVIS: In vivo imaging system
KEGG: Kyoto Encyclopedia of Genes and Genomes
LAD: Left anterior descending coronary artery
LDH: Lactate dehydrogenase
MDA: Lipid peroxidation Malondialdehyde
MPS: Mononuclear phagocyte system
PAI: Plasminogen activator inhibitors
PC: Protocatechualdehyde
PCI: Percutaneous coronary intervention
PEI: Polyethyleneimine
PM: Platelet membrane
PT: Prothrombin time
PTPN: Platelet membrane coated pH-responsive tPA nanoparticles
PVDF: Polyvinylidene difluoride
ROS: Reactive oxygen species
SD rats: Sprague–Dawley rats
TEM: Transmission electron microscope
TPN: PH-responsive tPA nanoparticles
TT: Thrombin time
AMI: Acute myocardial infarction
PBA: Phenylboronic acid
tPA: Tissue plasminogen activator
