# Supplementary material for: Thrombus-specific/responsive biomimetic nanomedicine for spatiotemporal thrombolysis and alleviation of myocardial ischemia/reperfusion injury
Source: J Nanobiotechnology. 2022 Dec 13;20:531. doi: 10.1186/s12951-022-01686-1 (PMC9749152; doi:10.1186/s12951-022-01686-1)
Supplement: Supplementary file 1 — Additional file 1: Figure S1. The purity of primary cardiomyocytes. Figure S2. TEM images of TPN in different pH conditions. Figure S3. Hydrodynamic size curves of PM (a), and PTPN in pH 7.4 (b) and pH 6.4 pure water solution (c). Figure S4: Activity of free tPA and PTPN to convert plasminogen to plasmin after 24 hour incubation. Figure S5. Cell viability of HUVECs treated with tPA (a) and PM (b) at different concentrations. Table S1. Routine blood routine analysis. Table S2. Coagulation function. Figure S6. The distribution of TPN and PTPN in different organs detected by IVIS. Figure S7. H&E staining images of organs harvested at 4 weeks post-injection of nanoparticles. Figure S8. Binding capacity of TPN, PM and PTPN to HUVECs detected by flow cytometry under hypoxia condition. Figure S9. H&E staining of cardiac thrombus with different treatments. Figure S10. ROS level of H9C2 under different treatments were detected by flow cytometry and MDA assay. Figure S11. Mitochondrial morphology and ROS level of H2O2 damaged HUVECs treated with PTPN. Figure S12. Mitochondrial membrane potential (JC-1) of HUVECs treated with PTPN. Figure S13. (a)TPM distribution of primary cardiomyocytes without treatment (Ctrl) or treated with H2O2 + PBS and H2O2 + PTPN. Figure S14. ROS level after different treatments with DHE staining. Figure S15. Fibrosis level after different treatments with PRS staining. Figure S16. Serum IL-1β (a) and TNF-α (b) levels in AMI rats with different treatments. [file 12951_2022_1686_MOESM1_ESM.docx]

**Thrombus-Specific/Responsive Biomimetic Nanomedicine for Spatiotemporal Thrombolysis and Alleviation of Myocardial Ischemia and Reperfusion Injury**

**Xiaoyu Guo^1, a, d^, Ting Hong^1, a, d^, Jie Zang^1, b^, Rongjiao Shao^a, d^, Xumin Hou^a^, Kai Wang^c^, Weizhuo Liu^a, d*^, Fan Su^a, d*^, Bin He^a, *^**

a. Department of Critical Care Medicine, Shanghai Chest Hospital, Shanghai Jiao Tong University, Shanghai, China.

b. The Institute for Biomedical Engineering & Nano Science, School of Medicine, Tongji University, Shanghai, China.

c. Central Laboratory, Shanghai Chest Hospital, Shanghai Jiao Tong University, Shanghai, China.

d. Center for Cardiopulmonary Translational Medicine, Shanghai Chest Hospital, Shanghai Jiao Tong University, Shanghai, China.

**Corresponding authors:** Bin He ([bin_he@sjtu.edu.cn](mailto:bin_he@sjtu.edu.cn)), Weizhuo Liu ([liuweizhuo@shsmu.edu.cn](mailto:liuweizhuo@shsmu.edu.cn)), Fan Su ([Sf2601@shchest.org](mailto:Sf2601@shchest.org)).

The PDF file includes:

1. Materials and Reagents
2. Supplementary Table S1-2
3. Supplementary Figure S1-16

**Materials and Reagents**

| Reagents | Company | Catalog No. |
| --- | --- | --- |
| Anti-CD34 | Abcam | ab81289 |
| Anti-CD42b | Proteintech | 12860-1-AP |
| anti-CD61 | Abcam | ab119992 |
| α-actinin | Miltenyi Biotec | 130-119-806 |
| GAPDH | Abcam | ab8245 |
| OxPhos Rodent WB Antibody Cocktail | Invitrogen | 45-8099 |
| MitoTracker™ Red CMXRos | Invitrogen | M7512 |
| Hoechst 34850 | Invitrogen | H21486 |
| JC-1 | Invitrogen | M34152 |
| DCFH-DA | Beyotime | S0033M |
| Dil | Beyotime | C1036 |
| fluorescein isothiocyanate (FITC) | Invitrogen | F1907 |
| Hematoxylin and Eosin Staining Kit | Beyotime | C0105S |
| Protein Silver Stain Kit | Yeasen | 36244ES30 |
| WGA | Sigma | L4895 |
| Fetal bovine serum | Gibco | 10100147 |
| HyClone Dulbecco's Modified Eagle Medium (DMEM) with high glucose | Cytiva | SH30022.01 |
| HyClone Dulbecco's Modified Eagle Medium (DMEM) with low glucose | Cytiva | SH30021.01 |
| 0.25% Trypsin-EDTA | Gibco | 25200072 |
| 0.25% Trypsin | Gibco | 15050057 |
| PBS without calcium, magnesium | Cytiva | SH30256.01 |
| HBSS without calcium and magnesium | Cytiva | SH30588.0 |
| PGE1 | Merck | P8908 |
| EDTA | Merck | E6758 |
| Complete Protease Inhibitor Cocktail | Merck | 04693116001 |
| PhosSTOP™ | Merck | 04906845001 |
| Complete Lysis-M buffer | Roche | 04719956001 |
| SDS-PAGE Gel Fast Preparation Kit | Epizyme | PG111, PG112, PG113 |
| CellTiter-Glo Luminescent Cell Viability Assay | Promega | G7570 |
| tPA (Mammalian, C-6His) | Novoprotein | CX67 |
| Plasmin Activity Assay Fluorometric | Abcam | ab204728 |
| PVDF membrane | Roche | 03010040001 |
| Avanti® Mini-Extruder | Merck | 610000 |

**Figure S1-S16**


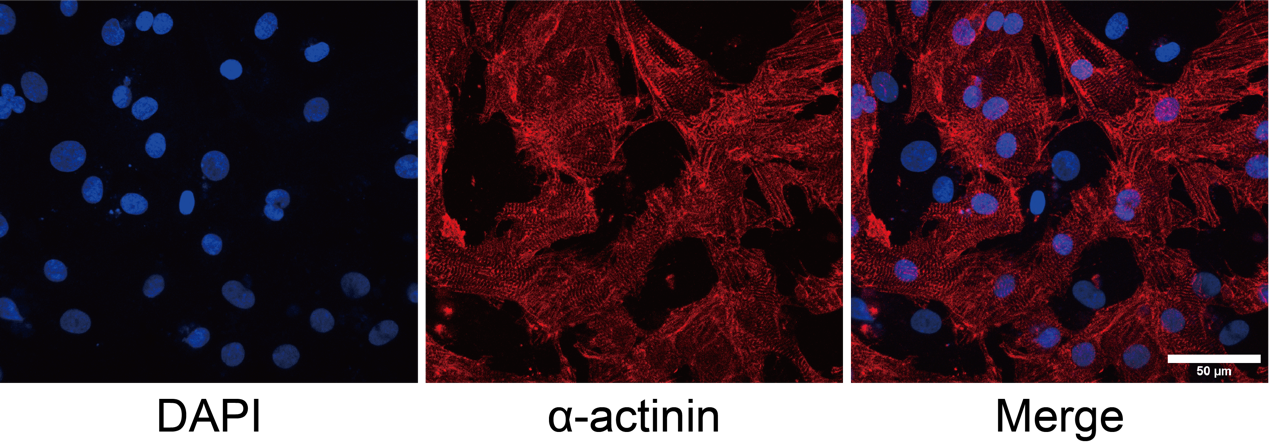


**Supplementary Figure S1:** The purity of primary cardiomyocytes. Primary cardiomyocytes were identified by the expression of α-actinin (red). Scale bar=50 μm.


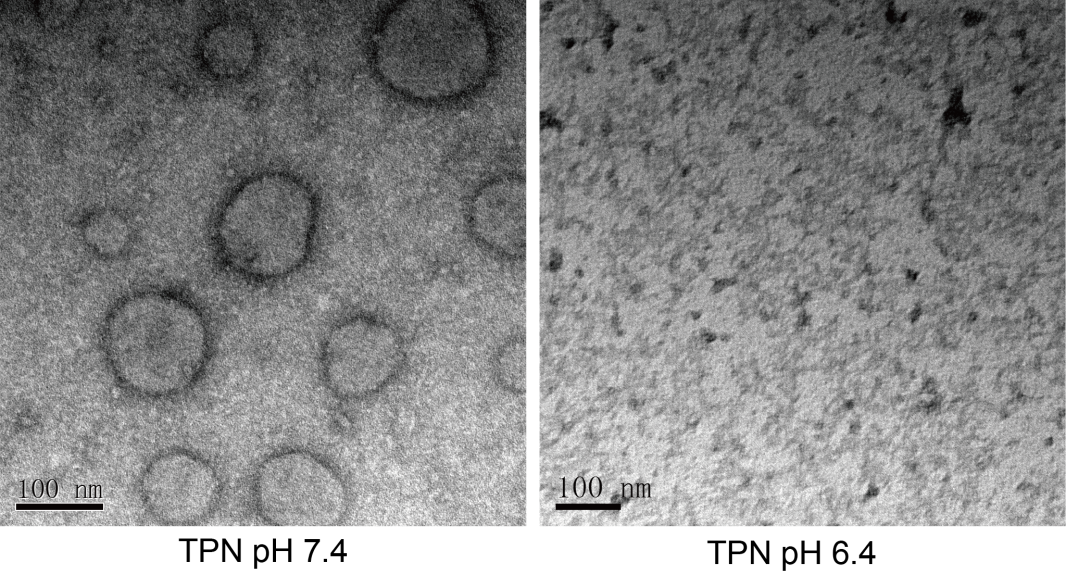


**Supplementary Figure S2:** TEM images of TPN in different pH conditions. Scale bar =100 nm.


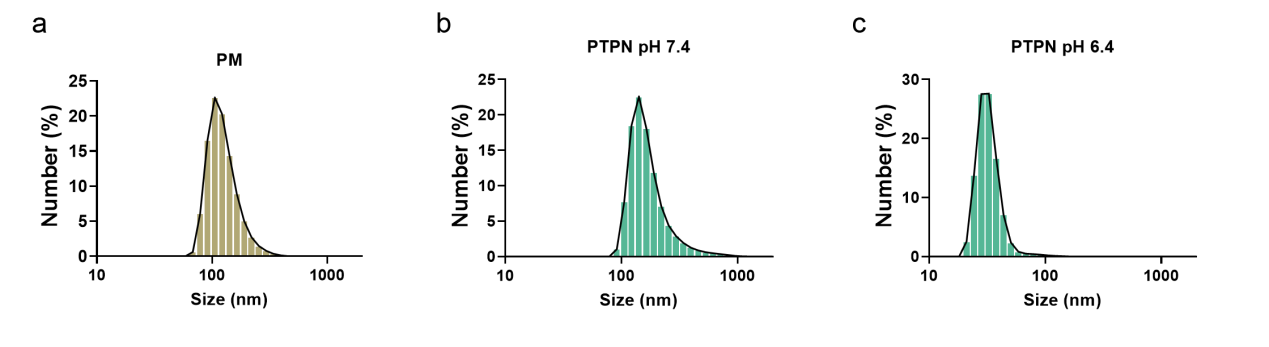


**Supplementary Figure S3:** Hydrodynamic size curves of PM (a), and PTPN in pH 7.4 (b) and pH 6.4 pure water solution (c).


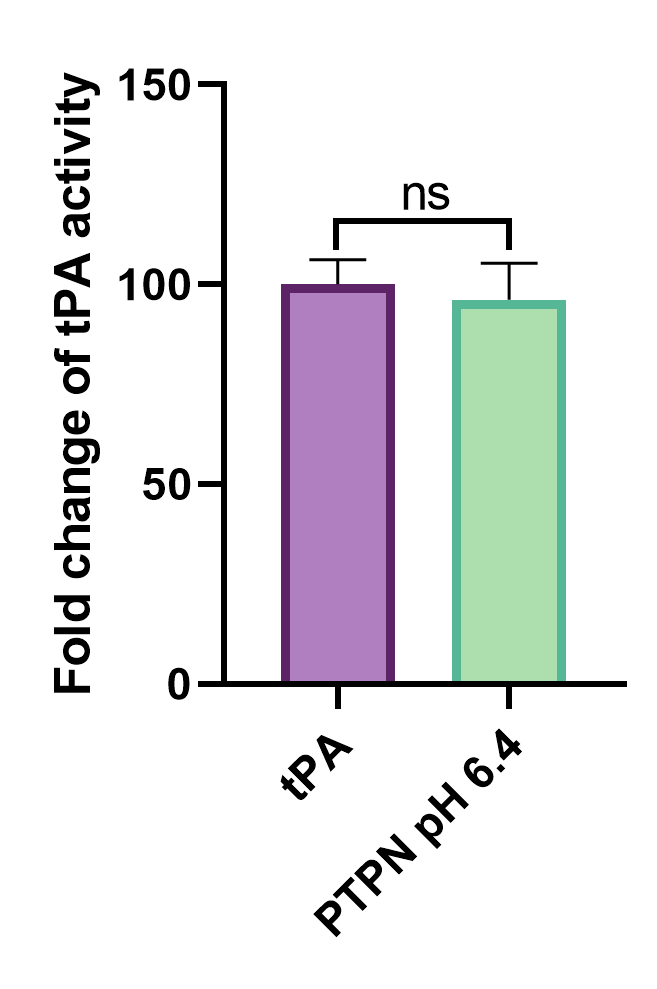


**Supplementary Figure S4:** Activity of free tPA and PTPN to convert plasminogen to plasmin after 24 hour incubation. (n=3).


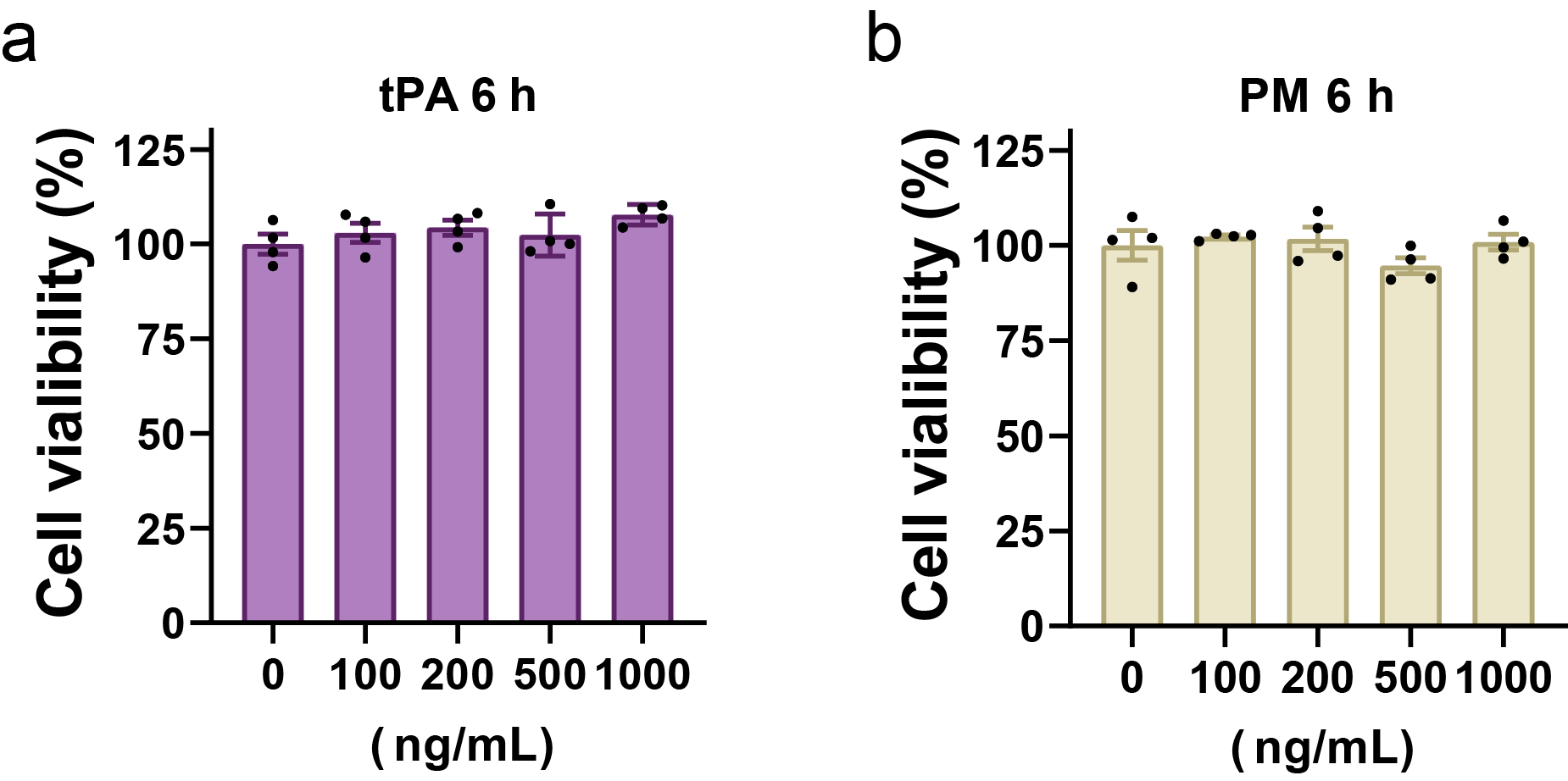


**Supplementary Figure S5:** Cell viability of HUVECs treated with tPA (a) and PM (b) at different concentrations (n = 4).

**Supplementary Table S1：**Routine blood routine analysis.

| Parameter | Abbreviation | Reference range | Unit | Ctrl | rt-PA | TPN | PM | PTPN |
| --- | --- | --- | --- | --- | --- | --- | --- | --- |
| total white blood cells | WBC | 0.8-6.8 | 10^9/L | 6 | 4.8 | 5.7 | 4.1 | 5.9 |
| lymphocyte count | Lymph# | 0.7-5.7 | 10^9/L | 4.2 | 3.36 | 3.81 | 2.829 | 3.98 |
| monocyte count | Mon# | 0.0-0.3 | 10^9/L | 0.23 | 0.18 | 0.22 | 0.21 | 0.28 |
| neutrophil count | Gran# | 0.1-1.8 | 10^9/L | 1.14 | 1.26 | 1.66 | 1.06 | 1.64 |
| the percentage of the lymphocyte | Lymph% | 55.8-90.6 | % | 70 | 70 | 66.9 | 69 | 67.5 |
| the percentage of the monocyte | Mon% | 1.8-6.0 | % | 3.8 | 3.7 | 3.9 | 5.2 | 4.7 |
| the percentage of the neutrophil | Gran% | 8.6-38.9 | % | 26.2 | 26.3 | 29.2 | 25.8 | 27.8 |
| red blood cell count | RBC | 6.36-9.42 | 10^12/L | 7.03 | 7.93 | 7.74 | 7.77 | 7.47 |
| hemoglobin | HGB | 110-143 | g/L | 132 | 144 | 131 | 149 | 136 |
| packed red blood cell volume | HCT | 34.6-44.6 | % | 40.2 | 44.3 | 41.2 | 44.6 | 44.5 |
| mean corpuscular volume | MCV | 48.2-58.3 | fL | 57.3 | 55 | 51.7 | 57.5 | 59 |
| mean corpuscular hemoglobin | MCH | 15.8-19 | pg | 18.7 | 18.1 | 16.9 | 19.1 | 18.2 |
| mean corpuscular hemoglobin concentration | MCHC | 302-353 | g/L | 328 | 329 | 327 | 334 | 324 |
| red blood cell volume distribution width | RDW | 13-17 | % | 13.2 | 13.1 | 11.2 | 13.5 | 10.9 |
| platelet count | PLT | 450-1590 | 10^9/L | 1348 | 1304 | 1355 | 1338 | 1199 |
| mean platelet volume | MPV | 3.8-6.0 | fL | 7.1 | 7.3 | 7.4 | 7.1 | 7.3 |
| platelet distribution width | PDW |  |  | 16.6 | 16.5 | 16.3 | 16.7 | 16.4 |
| thrombocytocrit | PCT |  | % | .*** | .*** | .*** | .*** | .*** |

**Supplementary Table S2：coagulation function.**

|  | PBS | rt-PA | PM | TPN | PTPN |
| --- | --- | --- | --- | --- | --- |
| PT（s） | 16.2 | 17.4 | 16.9 | 18.0 | 17.9 |
| APTT（s） | 40.7 | 58.3 | 42.0 | 53.7 | 40.9 |
| TT（s） | 42.5 | 42.3 | 44.0 | 42.0 | 39.8 |
| FIB（g/L） | 1.4 | 2.5 | 3.7 | 3.1 | 4.8 |


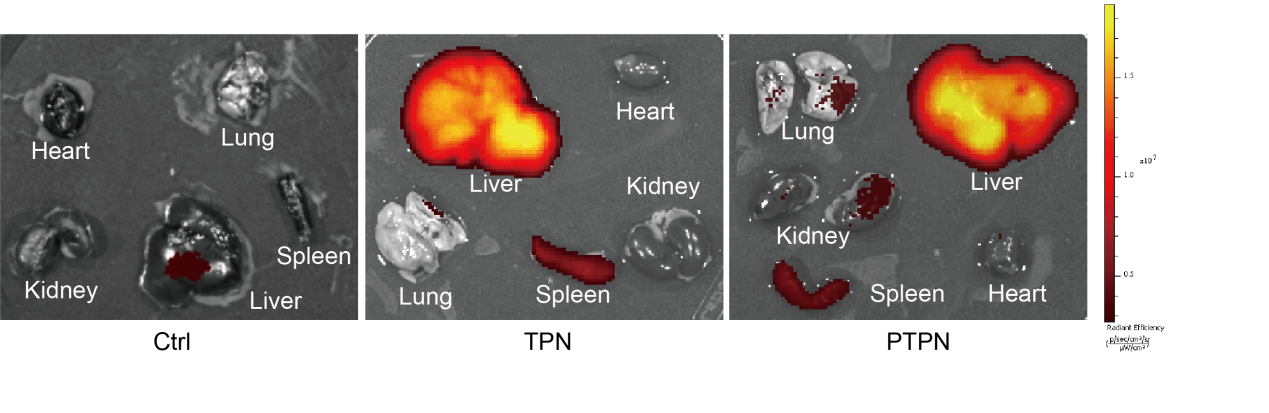


**Supplementary Figure S6:** The distribution of TPN and PTPN in different organs detected by IVIS.


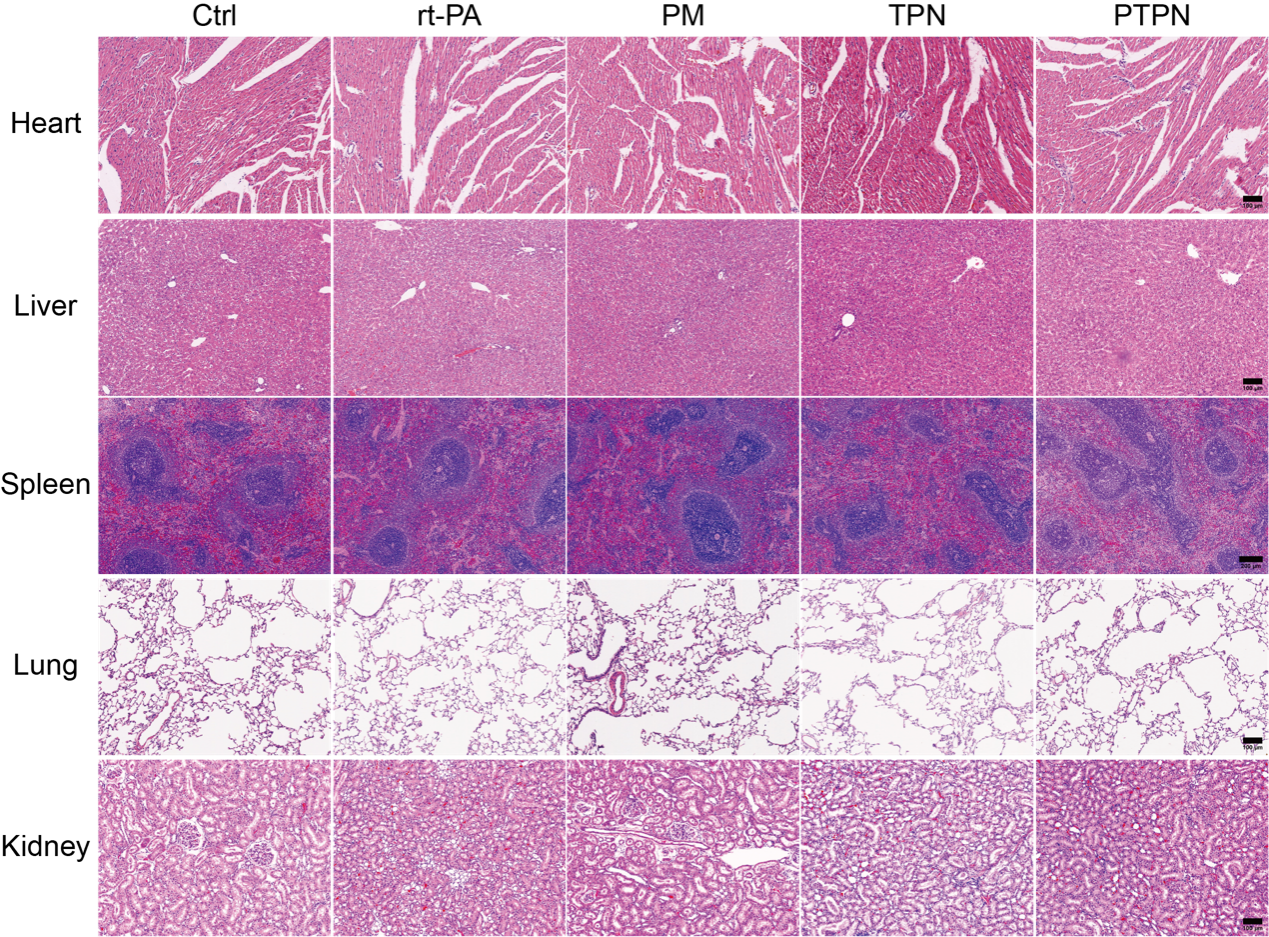


**Supplementary Figure S7:** H&E staining images of organs harvested at 4 weeks post-injection of nanoparticles. Scale bar = 100 µm.


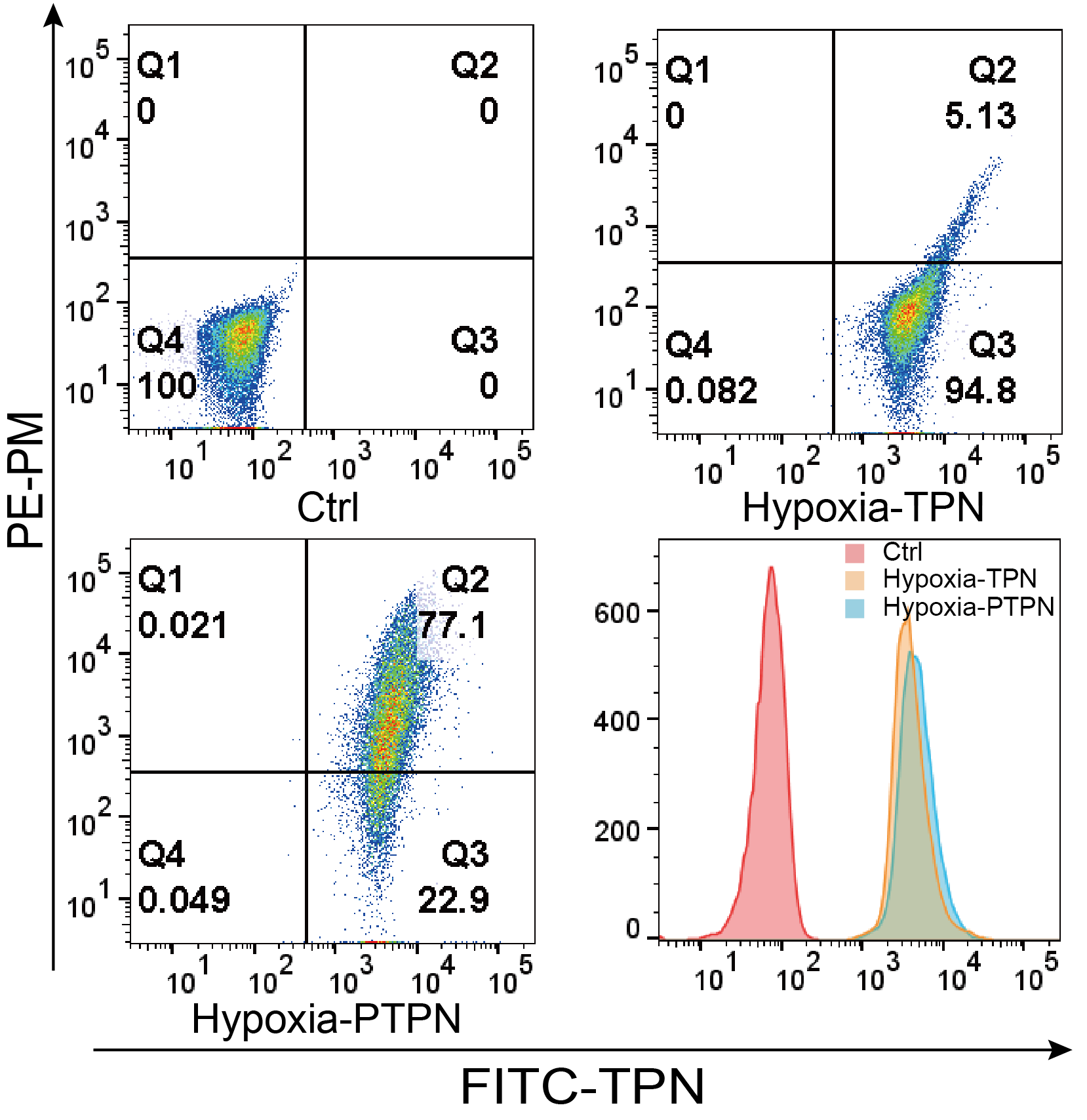


**Supplementary Figure S8:** Binding capacity of TPN, PM and PTPN to HUVECs detected by flow cytometry under hypoxia condition.


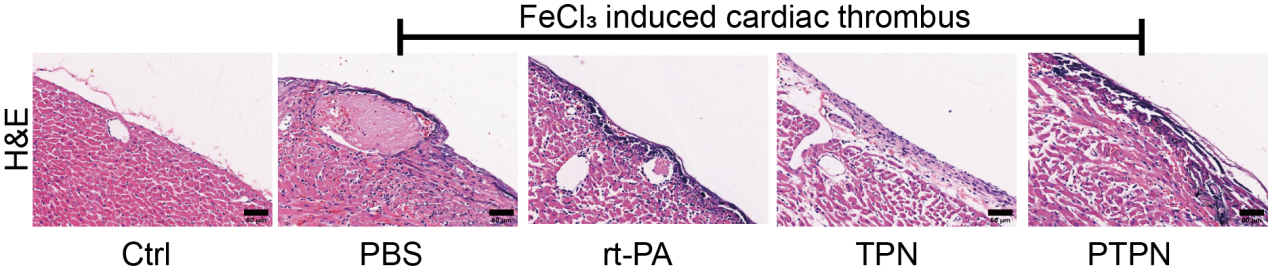


**Supplementary Figure S9:** H&E staining of cardiac thrombus with different treatments (n = 3). Scale bar = 50 μm.


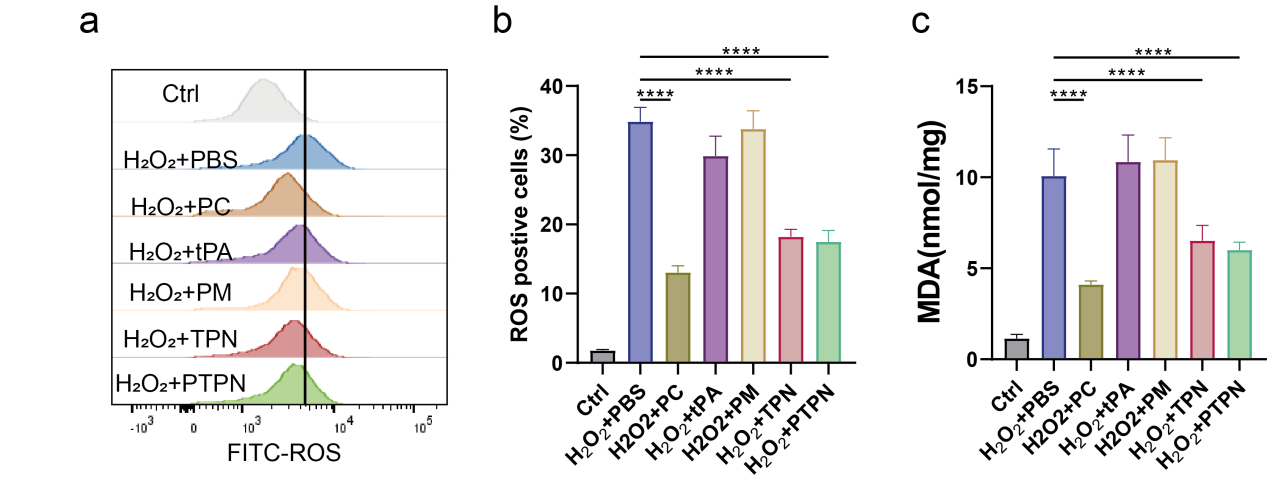


**Supplementary Figure S10：**ROS level of H9C2 under different treatments were detected by flow cytometry. (b) Quantitative analysis of ROS level (n=3). (c) Level of MDA in H9C2 cell under different treatment (n=3).


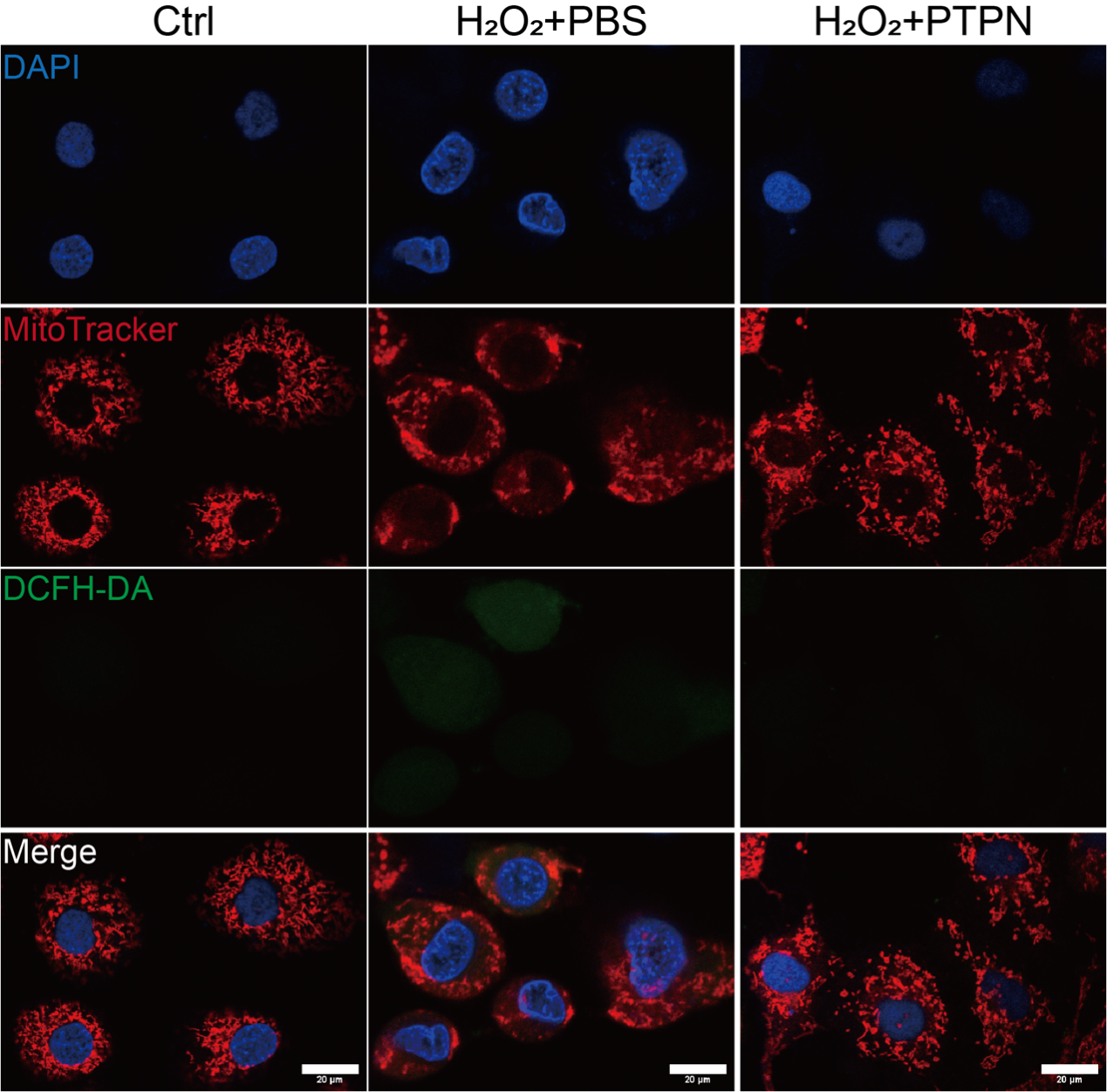


**Supplementary Figure S11:** Mitochondrial morphology (MitoTracker, red) and ROS level (DCFH-DA, green) of H_2_O_2_ damaged HUVECs treated with PTPN (n = 3; scale bar = 20 μm).


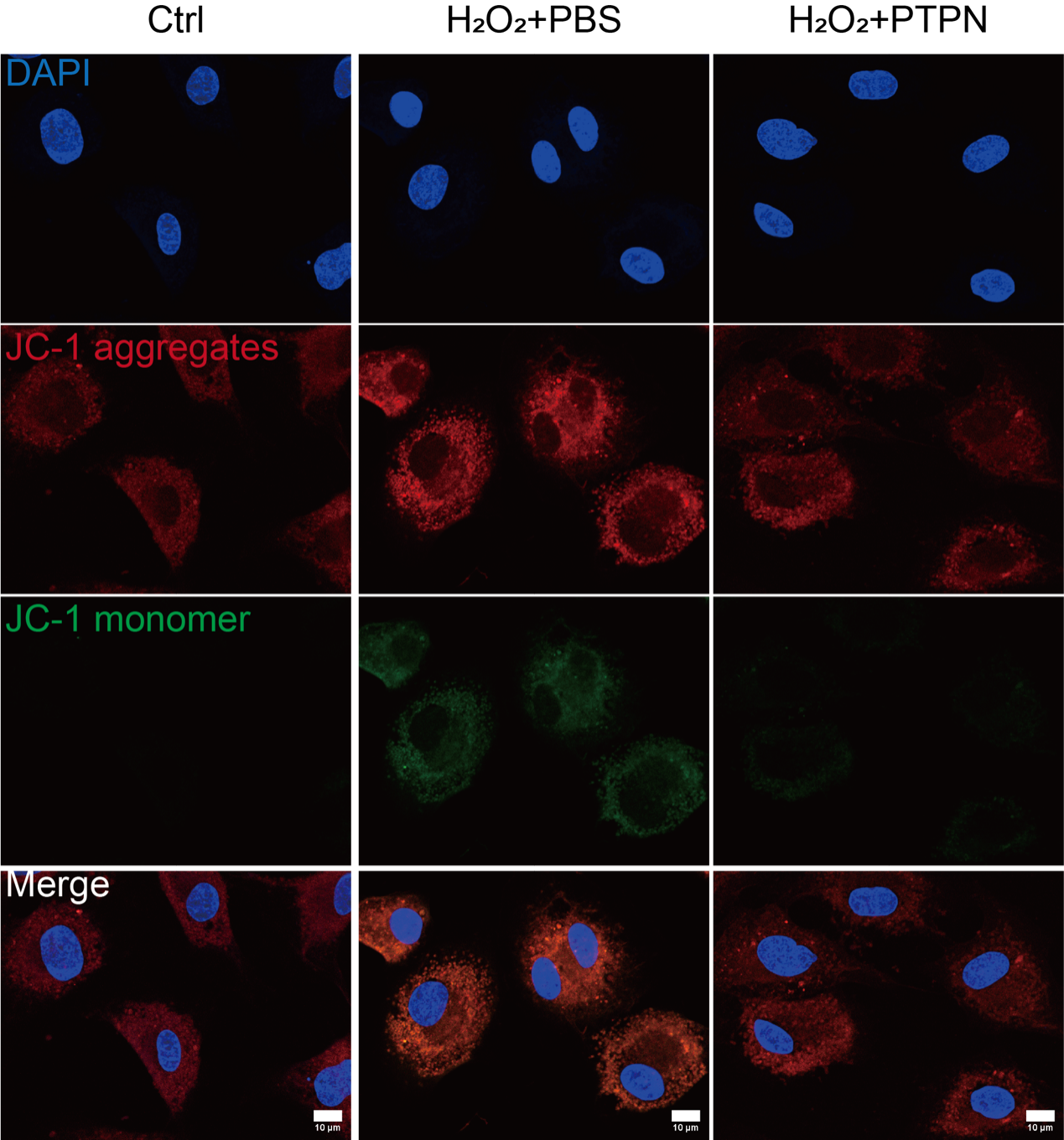


**Supplementary Figure S12:** Mitochondrial membrane potential (JC-1) of HUVECs treated with PTPN. Red indicated JC-1 aggregates and green indicated JC-1 monomer. (n = 3, scale bar = 20 μm).


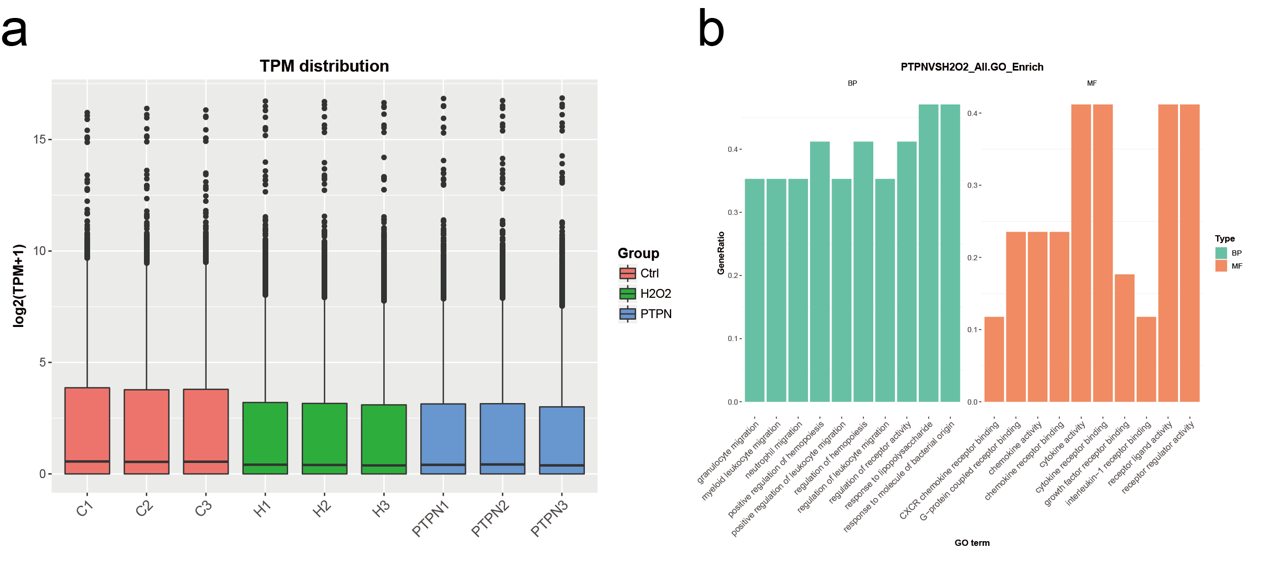


**Supplementary Figure S13:** (a)TPM distribution of primary cardiomyocytes without treatment (Ctrl) or treated with H_2_O_2_ + PBS and H_2_O_2_ + PTPN. (b) GO analysis of H_2_O_2_ + PBS VS H_2_O_2_ + PTPN.


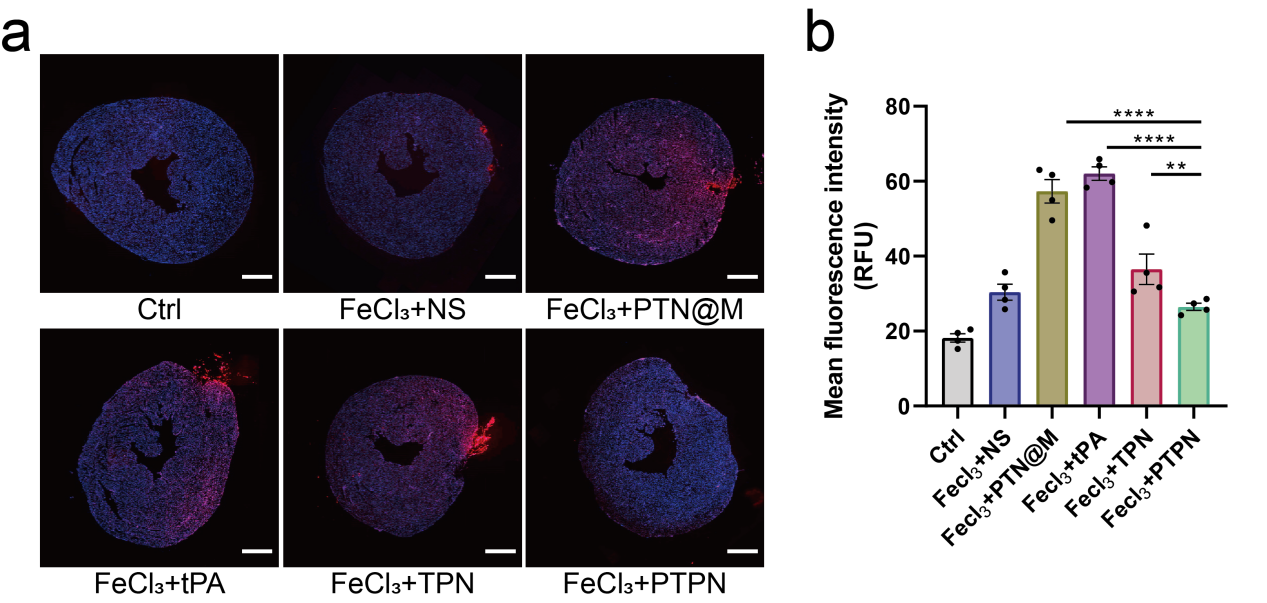


**Supplementary Figure S14:** (a) Representative photographs of ROS level after different treatments with DHE staining (n = 4); Scale bar = 1 mm. (b) Quantitative analysis of ROS level. Statistical analysis was via one-way ANOVA with GraphPad Prism 8.0; **p < 0.01 and ****p < 0.0001.


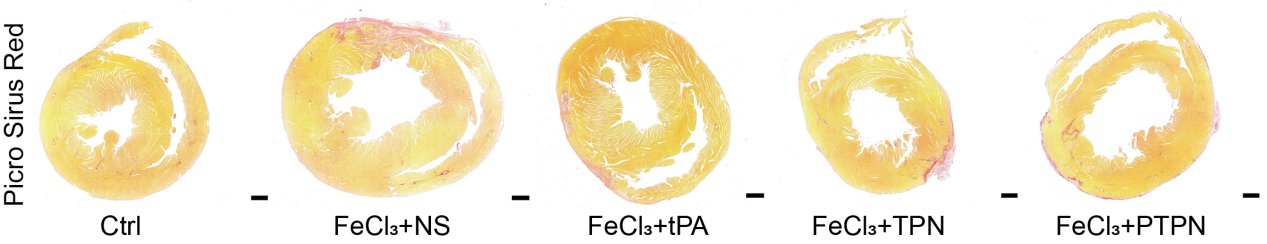


**Supplementary Figure S15:** Representative photographs of fibrosis level after different treatments with PRS staining (n = 4); Scale bar = 1 mm.


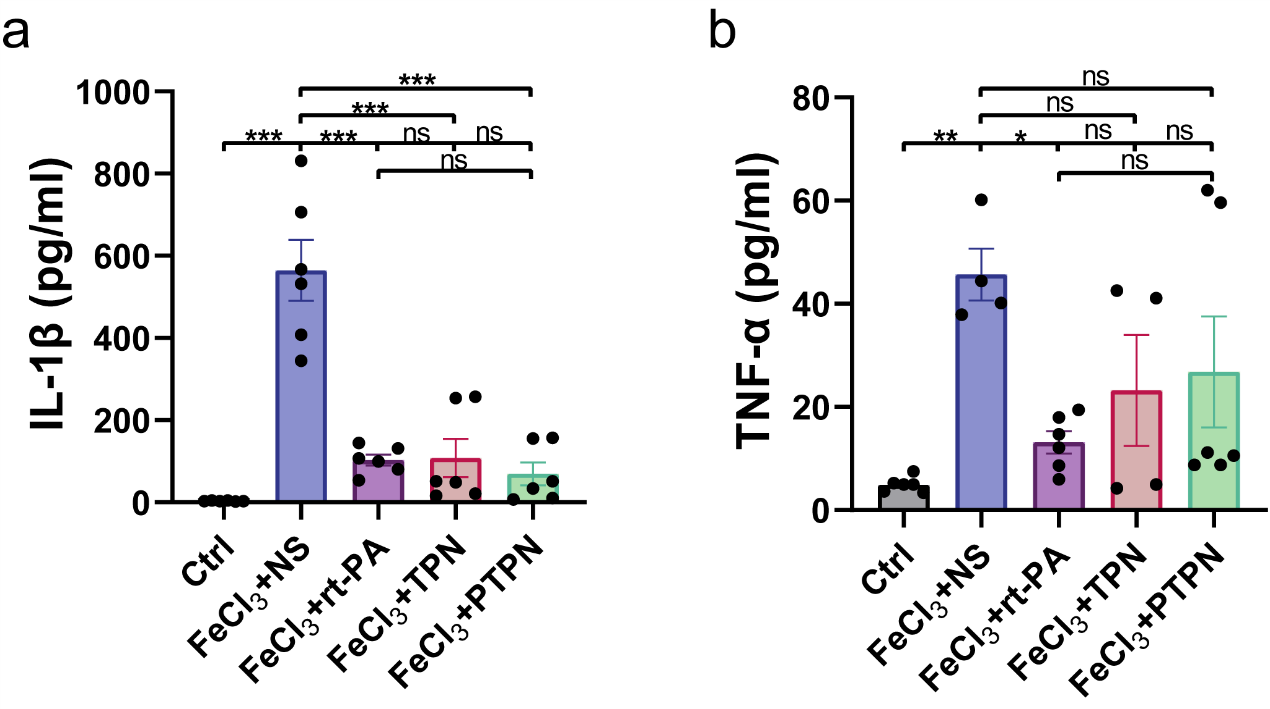


**Supplementary Figure S16:** Serum IL-1β (a) and TNF-α (b) levels in AMI rats with different treatments (n = 6). Data are presented as mean ± SEM. Statistical analysis was performed via one-way ANOVA with Bonferroni multiple-comparison correction using GraphPad Prism 8.0; ns: not significant; *p < 0.05; **p < 0.01 and ***p < 0.001.
